# Genome-wide MNase hypersensitivity assay unveils distinct classes of open chromatin associated with H3K27me3 and DNA methylation in *Arabidopsis thaliana*

**DOI:** 10.1186/s13059-020-1927-5

**Published:** 2020-02-03

**Authors:** Hainan Zhao, Wenli Zhang, Tao Zhang, Yuan Lin, Yaodong Hu, Chao Fang, Jiming Jiang

**Affiliations:** 1 Department of Plant Biology, Michigan State University, East Lansing, MI 48824 USA; 2 Department of Horticulture, Michigan State University, East Lansing, MI 48824 USA; 3 Department of Horticulture, University of Wisconsin-Madison, Madison, WI 53706 USA; 4 State Key Laboratory for Crop Genetics and Germplasm Enhancement, Nanjing Agriculture University, Nanjing, 210095 Jiangsu China; 5 Key Laboratory of Crop Genetics and Physiology of Jiangsu Province/Key Laboratory of Plant Functional Genomics of Ministry of Education, Yangzhou University, Yangzhou, 225009 China; 6 Department of Animal Sciences, University of Wisconsin-Madison, Madison, WI 53706 USA; 7 Michigan State University AgBioResearch, East Lansing, MI 48824 USA

**Keywords:** Open chromatin, *cis*-regulatory elements, Histone modification, DNA methylation, DNase-seq, ATAC-seq, *Arabidopsis thaliana*

## Abstract

**Background:**

Regulation of transcription depends on interactions between *cis*-regulatory elements (CREs) and regulatory proteins. Active CREs are imbedded in open chromatin that are accessible to nucleases. Several techniques, including DNase-seq, which is based on nuclease DNase I, and ATAC-seq, which is based on transposase Tn5, have been widely used to identify genomic regions associated with open chromatin. These techniques have played a key role in dissecting the regulatory networks in gene expression in both animal and plant species.

**Results:**

We develop a technique, named MNase hypersensitivity sequencing (MH-seq), to identify genomic regions associated with open chromatin in *Arabidopsis thaliana*. Genomic regions enriched with MH-seq reads are referred as MNase hypersensitive sites (MHSs). MHSs overlap with the majority (~ 90%) of the open chromatin identified previously by DNase-seq and ATAC-seq. Surprisingly, 22% MHSs are not covered by DNase-seq or ATAC-seq reads, which are referred to “specific MHSs” (sMHSs). sMHSs tend to be located away from promoters, and a substantial portion of sMHSs are derived from transposable elements. Most interestingly, genomic regions containing sMHSs are enriched with epigenetic marks, including H3K27me3 and DNA methylation. In addition, sMHSs show a number of distinct characteristics including association with transcriptional repressors. Thus, sMHSs span distinct classes of open chromatin that may not be accessible to DNase I or Tn5. We hypothesize that the small size of the MNase enzyme relative to DNase I or Tn5 allows its access to relatively more condensed chromatin domains.

**Conclusion:**

MNase can be used to identify open chromatin regions that are not accessible to DNase I or Tn5. Thus, MH-seq provides an important tool to identify and catalog all classes of open chromatin in plants.

**Electronic supplementary material:**

**Supplementary information** accompanies this paper at 10.1186/s13059-020-1927-5.

## Introduction

Complex biological processes such as cell differentiation and response to environmental cues rely on precise temporal and spatial control of gene transcription, which is governed by interactions between transcription factors (TFs) and *cis*-regulatory elements (CREs) [1–3]. Deciphering CREs in the genome is essential to understanding the transcription regulatory network which manifests tissue complexity and phenotypic polymorphism. Genomic regions containing active CREs are accessible to regulatory proteins via eviction or unraveling of nucleosomes in local chromatin [4, 5]. The accessibility, which is related to “open” or “closed” chromatin conformation, can be assayed by several technologies including DNase-seq and ATAC-seq [6–9]. In these methods, chromatin is treated by a small dose of endonuclease DNase I or transposase Tn5. Open chromatin that lacks protection of nucleosomes is preferentially attacked by these enzymes, which resulted in small DNA fragments associated with regulatory proteins. These DNA fragments can be identified by high-throughput sequencing. DNase-seq and ATAC-seq have been widely used for identification of CREs associated with different cell types, tissues, and developmental stages in both animals [10, 11] and plant species [12–18].

Micrococal nuclease (MNase) generates double-strand breaks at unprotected DNA and “nibbles” the exposed DNA until it encounters an obstruction, such as a nucleosome [19, 20]. Since linker DNA is preferentially attacked by MNase, chromatin treated with MNase would be digested into a nucleosomal ladder and eventually result in nucleosome cores protected by ~ 147 bp DNA [19, 21]. Given this unique property, MNase has mainly been used to investigate genome-wide nucleosomal occupancy and positioning, in which DNA fragments from 150 to 200 bp, which represent nucleosome footprints, are analyzed. Besides nucleosome footprints, shorter (< 80 bp) fragments associated with other DNA-binding proteins, such as TFs, in the MNase-treated chromatin were reported [20, 22]. Indeed, coupling MNase digestion with chromatin immunoprecipitation (ChIP) using antibodies against TFs has led to the identification of TF-binding sites with increased specificity and sensitivity than conventional protocols [23, 24]. These studies suggest the potential of MNase to decipher the regulatory landscape of eukaryotic genomes.

We developed a technique, named MNase hypersensitivity sequencing (MH-seq), to identify genomic regions associated with open chromatin in *Arabidopsis thaliana*. Briefly, the *A. thaliana* chromatin was fixed and lightly digested by MNase. The resulting small DNA fragments (20 to 100 bp) were collected and sequenced using the Illumina platform. Genomic regions enriched with MH-seq reads are referred as MNase hypersensitive sites (MHSs). We found that MHSs cover the majority (87–92%) of the open chromatin identified previously by DNase-seq and ATAC-seq. Surprisingly, a significant proportion (22%) of MHSs were not covered by DNase-seq or ATAC-seq reads, which are thereafter referred to “specific MHSs” (sMHSs). We demonstrate that sMHSs are enriched for H3K27me3 and DNA methylation and represent distinct classes of open chromatin domains that may not be accessible to DNase I or Tn5. sMHSs showed a number of distinct characteristics compared to the MHSs covered by DNase-seq or ATAC-seq reads, including association with transcriptional repressors. Thus, MH-seq provides a new tool to identify and catalog all classes of open chromatin in higher eukaryotes.

## Results

### Identification of open chromatin based on hypersensitivity to MNase

**Fig. 1:**
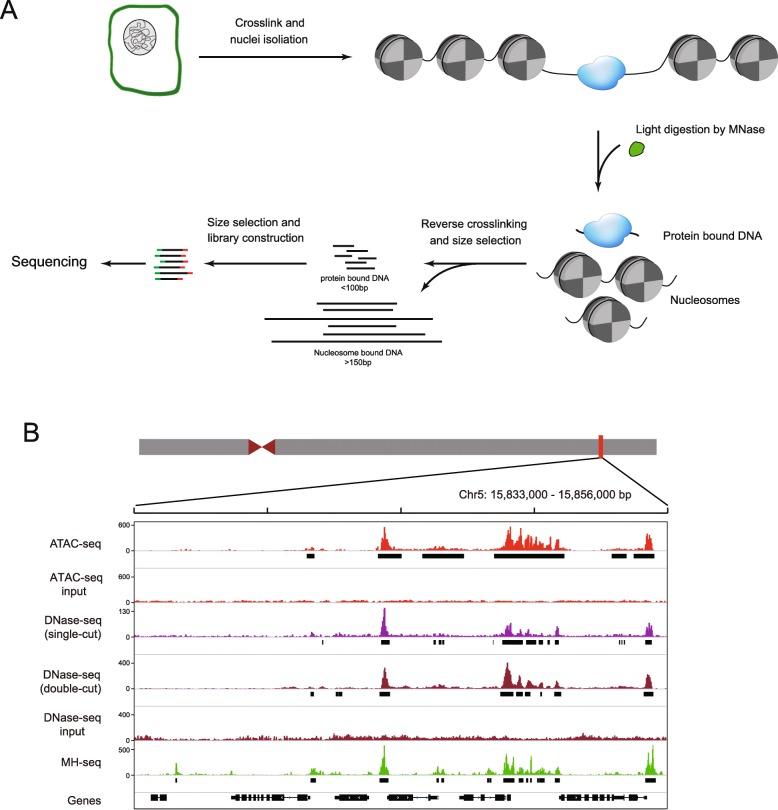
Identification of open chromatin based on MH-seq. **a** A schematic diagram of MH-seq. Chromatin is lightly digested by MNase. MNase cuttings at linkers generate large fragments (> 150 bp) which are protected by nucleosomes. MNase cutting at open chromatin regions generates small fragments (< 100 bp) which are associated with regulatory proteins. The small DNA fragments are collected for library construction and sequencing. **b** A representative open chromatin region on chromosome 5 based on MH-seq. Profiles from ATAC-seq and DNase-seq data (single-cut and double-cut) are included for comparison. Open chromatin regions are marked by black rectangle under profile of each technique. Gene models are shown at the bottom

We developed a MH-seq technique to recover small DNA fragments derived from genomic regions that are hypersensitive to MNase digestion (Fig. 1). These regions are referred as MNase hypersensitive sites (MHSs). Briefly, chromatin isolated from a target species, *A. thaliana* in the current study, was cross-linked using formaldehyde and then digested with a small amount of MNase (see “Methods”). For an appropriate level of digestion, the monomeric nucleosomal DNA band would represent the weakest band among the nucleosomal DNA bands in gel electrophoresis (Additional file 1: Figure S1). Small DNA fragments, ranging from 20 to 100 bp, were recovered from agarose gels for library construction and sequencing (MH-seq). The library is predicted to contain minimal amount of nucleosomal DNA (> 147 bp) and be enriched with sequences bound to regulatory proteins (Fig. 1). Linker-related DNA sequences in the library would be minimal because the monomeric nucleosomal DNA accounts only a small percentage of the total DNA (Additional file 1: Figure S1).

We developed a MHS library from 2-week-old seedling tissue of *A. thaliana* ecotype Col-0. Illumina sequencing of the library generated 111 million (M) of sequence reads. After adapter trimming and quality filtering, 110.7 M reads were used for further analyses. As expected, the majority of the sequence reads (90%) were of length of 30–60 bp (Additional file 1: Figure S1). We aligned 104 M reads to the *A. thaliana* reference genome (TAIR10) using Bowtie 2 [25]. We developed the software tool Jazz to identify MHSs, which represent genomic regions significantly enriched with MH-seq sequence reads. A single or multiple peaks were identified within each MHS by Jazz. We identified a total of 70,046 MHSs with 167,204 peaks in the *Arabidopsis* genome. Approximately 50% MHSs were located within 1 kb upstream of a transcription start site (TSS) and 1 kb downstream of transcription terminal site (TTS) (Additional file 1: Figure S2A). We then produced a biological replicate of the MH-seq data to test the reproducibility of the method. A total of 168 million reads were generated and 44,552 peaks were identified. The two data sets were highly correlated (*r* = 0.82, *p* value< 2.2 × 10–16). We found that 89.7% of the 44,552 peaks identified by the second dataset overlap with the original data although the second dataset has a higher percentage of reads mapped to chloroplast genome (48.4% versus 24.7%) and a higher background, which was measured by the fraction of reads in peaks (0.34 versus 0.61). Overall, MHSs and DNase I hypersensitive sites (DHSs) [13] shared a similar distribution pattern in *Arabidopsis* genome.

### MHSs span open chromatin containing *cis*-regulatory DNA sequences

We first used the genome-wide nucleosome positioning dataset from *A. thaliana* [26] to examine the level of nucleosome occupancy in regions associated with MHSs. We plotted the normalized read counts of nucleosomes within ± 1-kb regions surrounding the center of MHSs. As we expected, the MHS-associated genomic regions were depleted with nucleosomes (Additional file 1: Figure S3A).

To further confirm the association of MHSs with regulatory proteins, we examined the association of MHSs with genomic regions bound to different TFs. The TF-binding sites were previously detected by chromatin immunoprecipitation (ChIP) using seedling tissue and antibodies specific to each TF. The immunoprecipitated DNAs were used for microarray hybridization (ChIP-chip) or sequencing (ChIP-seq), which resulted in genome-wide binding datasets of 22 TFs [27] (Additional file 2: Table S1). We performed association analysis by comparing both MHS and DHS datasets with these 22 TF-binding datasets. We found that MHSs overlap with ≥ 75% of the binding sites from 18 of the 22 TFs (Additional file 1: Figure S2B). Overall, MHSs showed similar overlapping rates with TF-binding sites as peaks derived from DNase-seq and ATAC-seq datasets (Additional file 1: Figure S2B).

### Resolution of MHS-based detection of open chromatin

**Fig. 2:**
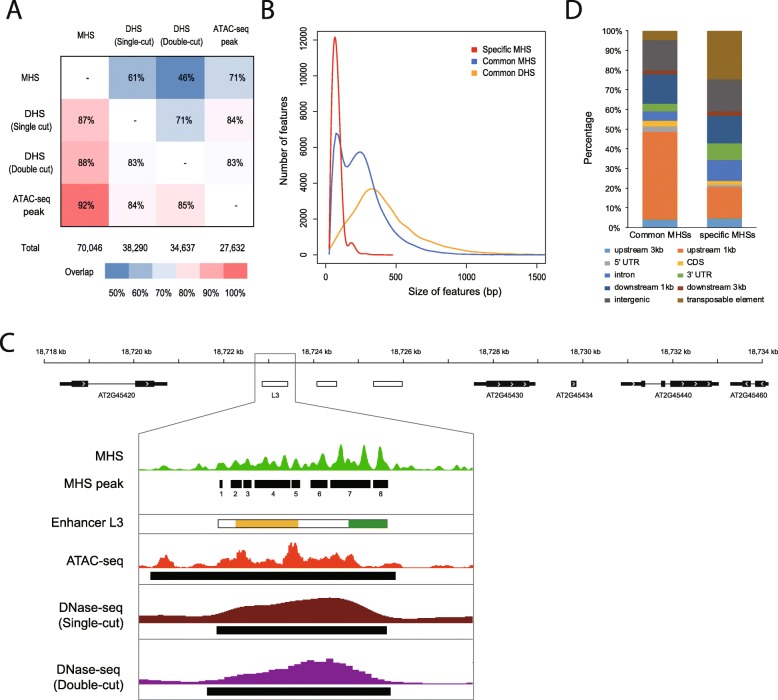
Characteristics of MHSs. **a** Overlaps among MHSs, DHSs, and ATAC-seq peaks. Percentage in each grid represents overlapping rate between the features in rows and the features in columns. The rows of the matrix represent the source features, while the columns represent the linked features. The percentage in each cell at the intersection of a row and column represents the overlapping rate between the source feature and the linked feature. For example, the cell at the intersection of MHS row and the DHS (single cut) column indicates that 61% of MHSs overlapped with DHSs. **b** Length distribution MHSs and DHSs. Specific MHSs, MHSs that were exclusively identified by MH-seq. Common MHSs: MHSs that are also identified by DNase-seq and ATAC-seq. Common DHSs: DHSs that are also identified by MH-seq and ATAC-seq. **c** MH-seq profile associated with an intergenic enhancer L3. L3 (chr2: 17722957–18723472) was previously identified by DNase-seq [28]. Gene models are shown at the top of the panel. Several small MH-seq peaks were identified within L3. White rectangles represent intergenic enhancers identified by DNase-seq. Orange and green rectangles mark regions associated with root- and leave-specific enhancer activity, respectively. Black rectangles represent open chromatin regions identified by MH-seq, DNase-seq and ATAC-seq. **d** Genomic features associated with cMHSs and sMHSs. “intergenic” represents the genomic regions that at least 3 kb away from genes but not overlap with transposable elements. “Transposable elements” represents genomic regions that at least 3 kb away from genes and overlap with transposable elements

We compared datasets from MH-seq, DNase-seq (based on nuclease DNase I) from both single-cut [13] and double-cut [14] libraries (see “Methods” for the difference of these two DNase-seq techniques), and ATAC-seq [15]. The peaks derived from DNase-seq and ATAC-seq techniques largely overlapped among each other (Fig. 2a), suggesting a similar capacity of these two techniques to detect open chromatin regions. Approximately 87–92% of the peaks identified by DNase-seq or ATAC-seq were covered by MHSs. We analyzed the correlation of peak levels of MH-seq, DNase-seq, and ATAC-seq. The MHSs that can be identified by both DNase-seq and ATAC-seq were used for comparison and the peak levels were measured as the reads per million [29]. We found that the peak levels of MH-seq, DNase-seq, and ATAC-seq were well correlated. The correlation of peak levels between MH-seq and DNase-seq is 0.38 (*p* value < 1 × 10–16, Spearman’s rank correlation coefficient); 0.25 between MH-seq and ATAC-seq (*p* value < 1 × 10–16, Spearman’s rank correlation coefficient); and 0.42 between DNase-seq and ATAC-seq (*p* value < 1 × 10–16, Spearman’s rank correlation coefficient).

We compared MHSs and single-cut DNase-seq data [13] as both datasets were developed from our lab using seedlings at the same development stage and grown under the same condition. We found that 38,290 (87%) DHSs overlapped with 42,678 (61%) MHSs. For regions identified by both DNase-seq and MH-seq, the average length of the MHSs was shorter than DHSs (Fig. 2b) (262 bp vs 423 bp, *p* value < 2.2 × 10^− 16^, Mann–Whitney *U* test). Furthermore, many MHSs contained multiple peaks with an average length of 47 bp (Additional file 1: Figure S3C). Most genomic regions detected by both MH-seq and DNase-seq were more narrowly spanned by MH-seq reads than by DNase-seq reads (Additional file 1: Figure S3B). Thus, MHSs showed a higher resolution than DHSs to detect open chromatin regions.

We analyzed the MHS that spans enhancer L3, which was previously identified by DNase-seq [28]. L3 is 625 bp in length and contains multiple regulatory elements which drive gene expression in both root and leaf tissues in reporter assays [28]. The single- and double-cut DNase-seq datasets identified this enhancer as a single peak. In contrast, the MH-seq revealed several peaks (Fig. 2c, Additional file 1: Figure S3C). Peaks 2, 3, 4, and 5 overlapped with a domain that shows enhancer activity in root tissue. Peaks 7 and 8 overlapped with a domain that shows enhancer activity in leaf tissue (Fig. 2c).

### MH-seq unveils open chromatin that may not be accessible to DNase I or Tn5

**Fig. 3:**
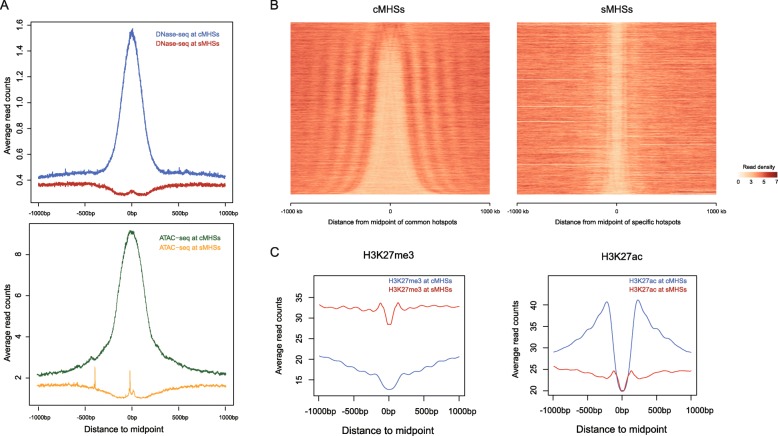
DNase I and Tn5 sensitivity and nucleosome occupancy associated with MHSs. **a** DNase I and Tn5 sensitivity at cMHSs and sMHSs. All cMHSs and sMHSs were aligned at the midpoints and the average DNase I sensitivity at each base pair spanning ± 1 kb from the midpoint was plotted. Blue and red line represent DNase I sensitivity of cMHSs and sMHSs, respectively. Green and orange lines represent Tn5 sensitivity at cMHSs and sMHSs, respectively. **b** Heatmaps of nucleosome occupancy spanning ± 1 kb from the midpoints of cMHSs and sMHSs, respectively. Nucleosome occupancy was calculated by deepTools. Each line represents nucleosome occupancy of one cMHS or sMHS. cMHSs and sMHSs were sorted by length in the plot. **c** Distribution of H3K27me3 and H3K27ac at MHSs. Genomic regions ± 1 kb from the midpoints of MHSs were divided into 10-bp bins and average number of H3K27me3 and H3K27ac reads in each bin were calculated

Surprisingly, 15,354 MHSs (22%) were not covered by DNase-seq or ATAC-seq reads, which are thereafter referred to “specific MHSs” (sMHSs, Additional file 1: Figure S3D, Additional file 1: Figure S4A-E). sMHSs tended to be located within intergenic regions (at least 3 kb away from any annotated genes, as well as those associated with TEs) (Fig. 2d) compared to the MHSs that are covered by DNase-seq or ATAC-seq reads, thereafter referred to “common MHSs” (cMHSs). Interestingly, inspecting the MHSs located in the intergenic regions revealed that 18% of the sMHSs were associated with transposable elements (TEs). In contrast, only 4% of cMHSs were associated with TEs. In addition, the average length of sMHSs is significantly shorter than that of cMHSs (average 85 bp vs 262 bp, Fig. 2b). Intriguingly, the DNase I and Tn5 sensitivity associated sMHSs was lower than cMHSs (Fig. 3a**)**, suggesting that the sMHS-associated chromatin domains were more resistant to DNase I and Tn5 digestion compared to those associated with cMHS. Despite these differences, sMHSs and cMHSs showed a similar distribution pattern along the chromosomes (Additional file 1: Figure S5). We analyzed the functions of genes associated with sMHSs and cMHSs, respectively. Comparing to genes cognate with cMHSs, genes associated with sMHSs were enriched in cell differentiation (FDR = 2.1 × 10^− 2^) and cellular developmental process (FDRe = 2.1 × 10^− 2^) and underrepresented in cellular process (FDR = 7.4 × 10^− 6^) and metabolic process (FDR = 2.1 × 10^− 4^).

We then analyzed the nucleosome occupancy associated with sMHSs and cMHSs, independently. A clear depletion of nucleosomes was observed at both cMHSs and sMHSs (Fig. 3b). The depletion of nucleosomes at cMHSs and sMHSs were further revealed by mapping of the H3 ChIP-seq data [30] (Additional file 1: Figure S3E). The cMHSs showed relatively wide and variable length of nucleosome-depleted regions (NDRs) flanked with well-positioned nucleosomes, which is similar to the nucleosome occupancy patterns around DHSs [31]. By contrast, sMHSs showed relatively narrow NDRs (Fig. 3b), which is consistent with the relatively short lengths of the sMHSs. Interestingly, sMHSs were not flanked with well-positioned nucleosomes (Fig. 3b). These results suggest that sMHSs may represent unique chromatin domains that are not accessible to DNase I and Tn5. Thus, these regions were not covered by DNase-seq and ATAC-seq reads.

### Distinct epigenomic features associated with sMHSs

We next compared the histone modification patterns associated with cMHSs and sMHSs, respectively. H3K27ac and H3K27me3 are antagonistic epigenetic marks, which are typically associated with active and inactive chromatin, respectively [32, 33]. Remarkably, cMHSs and sMHSs showed distinct difference of these two marks. The flanking regions of cMHSs were associated with a low level of H3K27me3 and high level of H3K27ac (Fig. 3c), similar to patterns associated with DHSs [12]. In contrast, sMHSs showed an opposite trend with a high level of H3K27me3 and a low level of H3K27ac (Fig. 3c). The sMHSs located in intergenic (*n* = 4080) and genic (*n* = 11,274) regions showed identical patterns (Additional file 1: Figure S6A), indicating that the distinct modifications of H3K27ac and H3K27me3 is an intrinsic feature for both sMHSs and cMHSs. In addition, sMHSs showed a higher level of DNA methylation relative to cMHSs (Additional file 1: Figure S6B).

**Fig. 4:**
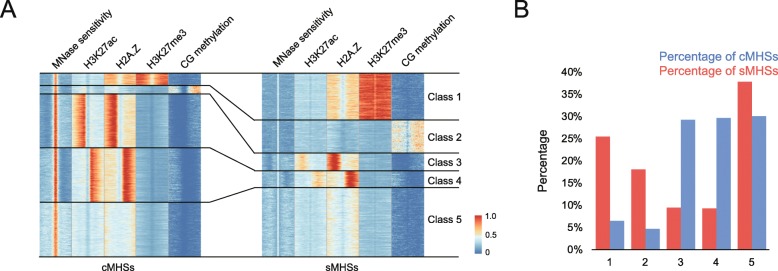
Clustering analysis of cMHSs and sMHSs. **a** Heatmaps of cMHSs and sMHSs generated by k-means cluster analysis using epigenetic marks H3K27ac, H2A.Z, H3K27me3, and CG DNA methylation. Genomic regions up- and downstream 1 kb of centers of MHSs were divided into 40 bins. The average read depth in each bin was calculated. The average read depth from each data set was then scaled from 0 to 1 and plotted as a heatmap. The five classes identified by k-mean cluster analysis are marked by black lines. **b** Percentage of cMHSs and sMHSs within each of the five classes

To further explore the chromatin characteristics associated with sMHSs and cMHSs, we performed K-means clustering of epigenetic marks H3K27ac, H2A.Z, H3K27me3, and DNA methylation. Five distinct clusters were identified based on the chromatin states of all MHSs (Fig. 4a, Additional file 1: Figure S7A). Class 1 was associated with repressive mark H3K27me3. Class 2 was associated with DNA methylation in CG, CHG, and CHH context at the flanking region of the MHSs (Additional file 1: Figure S7A). Classes 3 and 4 were enriched with asymmetric H3K27ac and H2A.Z and depleted of H3K27me3 and DNA methylation. Class 5 was associated with a low level of H2A.Z and H3K27ac. Classes 1, 2, and 5 tended to be located in intergenic regions while classes 3 and 4 were located at upstream of genes (Additional file 1: Figure S7A). We found that 26% and 18% of sMHSs were grouped into classes 1 and 2, which are associated with repressive epigenetic marks (Fig. 4b). In contrast, only 6% and 5% of cMHSs were grouped into these two classes. Inversely, a total of 18% of sMHSs were grouped to classes 3 and 4, whereas 59% of cMHSs were grouped to classes 3 and 4 (Fig. 4b). Thus, sMHSs were more frequently embedded within chromatin domains that are enriched with repressive epigenetic marks, including DNA methylation and H3K27me3.

### Functional analyses of sMHSs

We were intrigued by the potential function of sMHSs associated with H3K27me3 and DNA methylation. We examined the expression level and pattern of neighboring genes associated with sMHSs in classes 1 and 2. A sMHS-cognate gene was defined as the closest gene located within 1 kb up- and downstream of the sMHS. Genes associated with these sMHSs generally showed lower expression levels compared to genes associated with cMHSs (Additional file 1: Figure S8). We also analyzed gene expression profiles across 79 tissues [34]. Genes associated with class 1 sMHSs showed a high tissue specificity, suggesting that sMHSs associated with H3K27me3 may function mainly at specific development stages or cell types (Additional file 1: Figure S8). Consistently, gene ontology (GO) analysis showed that genes associated with class 1 sMHSs are enriched in developmental process (FDR = 1.6 × 10^− 12^), cell differentiation (FDR = 1.1 × 10^− 9^), and sequence-specific DNA-binding TF activity (FDR = 1.2 × 10^− 12^) when compared to genes associated with cMHSs. Interestingly, genes associated with class 2 sMHSs showed the same tissue specificity as cMHSs and no enriched function groups were identified after multiple testing correction (FDR < 0.01).

**Fig. 5:**
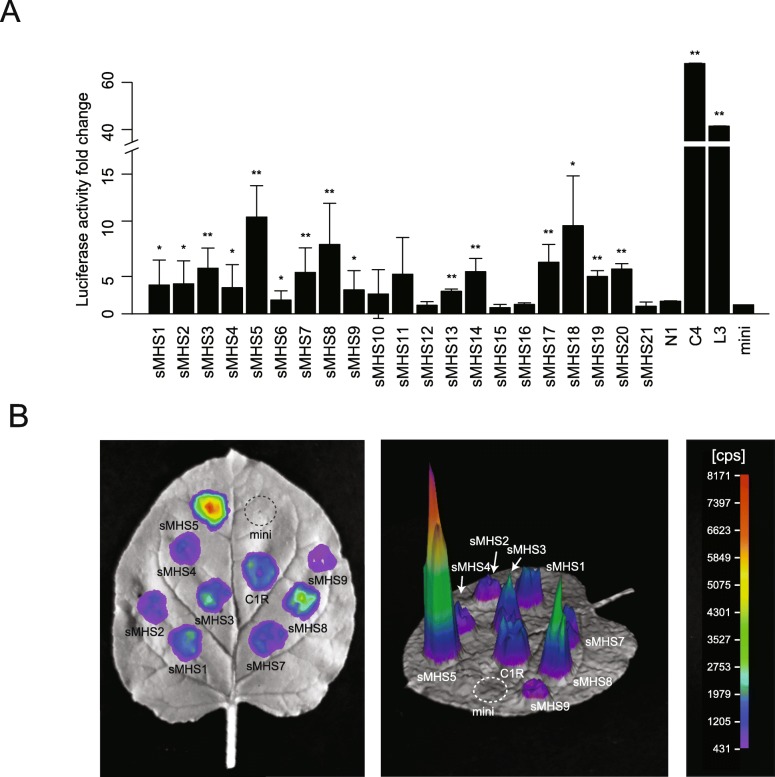
Functional validation of sMHSs using an agrobacterium-mediated transient assay. **a** The relative potential of 21 sMHSs in transcriptional regulation. *N. benthamiana* leaves were transiently transformed using *Agrobacteria* containing constructs of sMHS-mini35S::LUC. sMHS1-sMHS10 represent the 10 sMHSs associated with H3K27me3. sMHS11-sMHS21 represent the 11 sMHSs not associated with H3K27me3. L3 and C4 were validated as strong enhancers in leave, and N1 has no function to drive gene expression in a previous study [28]. *y*-axis represents the fold enrichment of luciferase signals of each construct compared to a construct containing only the mini35S promoter. **b** A representative *N. benthamiana* leaf infiltrated with different constructs. Color scale represents the luminescent signal intensity measured by cps (counts per second)

We designed experiments to examine the capacity of the sMHSs in driving gene expression*.* We randomly selected 10 sMHSs associated with H3K27me3 and 10 sMHSs not associated with H3K27me3 for experimental validation (Additional file 3: Table S2). The selected sMHSs are located in different genomic regions, including promoters, gene body region, or intergenic regions (Additional file 3: Table S2). The DNA sequences of these 20 sMHSs were synthesized and cloned into vector pCAMBIA-CRE-LUC, which contains a firefly luciferase reporter gene and the minimal cauliflower mosaic virus (CaMV) 35S promoter (− 50 to − 2 bp). The sMHSs were placed upstream of the mini35S promoter. The constructs were then infiltrated into *Nicotiana benthamiana* leaves using agrobacterium [35]. The transcription of the reporter gene would depend on if the sMHS can act as a *cis*-regulatory element, as the mini35S promoter alone cannot drive the transcription of the reporter gene [35]. Three replications of experiments were conducted for each construct. Nine sMHSs associated with H3K27me3 and six sMHSs not associated with H3K27me3-generated luciferase signals that were significantly higher than the mini35S promoter (Fig. 5), confirming the regulatory potential of sMHSs in gene expression.

### Function of sMHSs associated with H3K27me3 and DNA methylation

Both H3K27me3 and DNA methylation are commonly referred as repressive epigenetic marks. DNA methylation is a relatively stable epigenetic mark and is less dynamic compared to H3K27me3 during development, with special exceptions such as in the endosperm or gametes [36–38]. We speculated that the sMHSs associated with these marks may span repressed promoters or enhancers in 2-week seedling tissue and, thus, are “poised” to function in later developmental stages or in other tissues. To test this hypothesis, we analyzed chromatin accessibility of class 1 and 2 sMHSs in (1) *ddm1* mutant, which showed 50% decrease of DNA methylation [38], and in (2) flower buds, a different tissue compared to the 2-week seedling. We expected that some of these sMHSs are associated with an increased level of DNase I sensitivity if they become active in *ddm1* mutant or in flower buds.

**Fig. 6:**
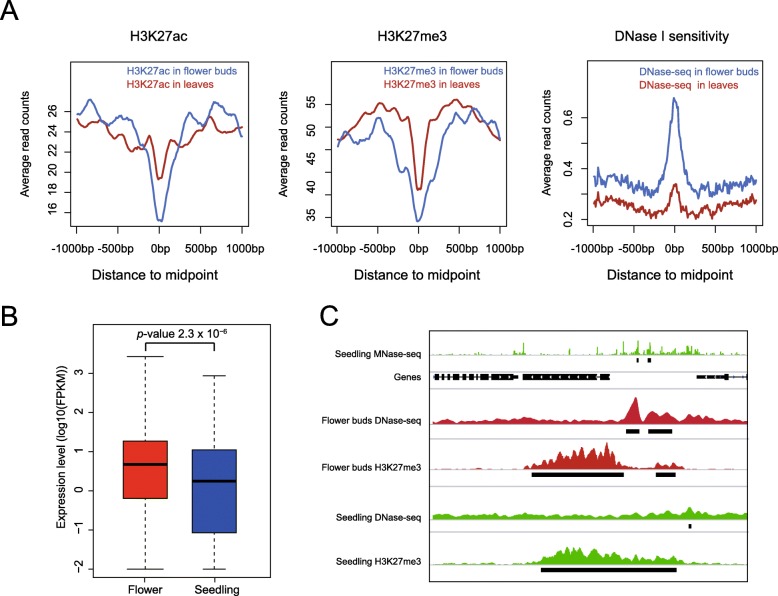
Functional analyses of sMHSs. **a** Distribution of H3K27me3, H3K27ac, and DNase I sensitivity at 382 sMHSs that can be detected by DNase-seq in leaves and flower buds. **b** Expression levels of genes associated with 382 sMHSs in leaves and flower buds. The cognate genes were defined as the closest genes located within 1 kb up- and downstream of MHSs. **c** A representative genomic region showing two sMHSs identified in seedling. These MHSs can be detected by DNase-seq in flower buds

We first examined if the class 1 sMHSs (with H3K27me3) can be detected by DNase-seq in flower buds. Indeed, 7.5% (382 of 5078) of the class 1 sMHSs were associated with DHSs (Fig. 6a). These sMHSs were also associated with a low level of H3K27me3 and a high level of H3K27ac in flower buds (Fig. 6a). Genes associated with these 382 sMHSs showed a higher expression level in flower buds than in seedling (Fig. 6b). For example, two sMHSs were detected at the promoter of AT5G58460. This gene is associated with H3K27me3 and is not expressed in seedling (FPKM = 0). In flower buds, the H3K27me3 at the promoter is not detectable. This gene is expressed in flower buds (FPKM = 2.3) and is associated with two DHSs detected at the same regions of the two sMHSs (Fig. 6c).

DNA methylation has a dynamic impact on gene expressions. DNA methylation at the gene body is frequently associated with active transcription while at promoter regions associated repression [39, 40]. Genes associated with class 2 sMHSs were found to be methylated at gene body only or both promoter and gene body (Additional file 1: Figure S9). Genes with exclusively body methylation were dominated by CG methylation. In contrast, genes with both promoter and body methylation were associated with all three contexts (CG, CHG, and CHH). Comparing the expression levels of genes associated with class 2 sMHSs between wild type and *ddm1* mutant showed that only few genes (94 out of 2970, 3.2%) changed the expression level in *ddm1* (Additional file 1: Figure S10A).

We analyzed whether the DNA methylation associated with class 2 MHSs was changed in *ddm1* by calculating the methylation levels of ±1 kb around sMHSs and cMHSs in wild type and *ddm1* mutant. We found that the DNA methylation levels of all the three contexts were reduced in the *ddm1* mutant, especially CG methylation (Additional file 1: Figure S10B). We then analyzed whether changes of DNA methylation altered the openness of these MHSs. Due to the lack of MH-seq data for *ddm1*, we analyzed the DNase I sensitivity of cMHSs in *ddm1* mutant. We found that the DNase I sensitivity of these cMHSs in *ddm1* were nearly unchanged (Additional file 1: Figure S10C). Thus, DNA methylation changes in flanking sequences did not alter the openness associated with class 2 MHSs.

### Transcription factors and DNA motifs associated with sMHSs and cMHSs

**Fig. 7:**
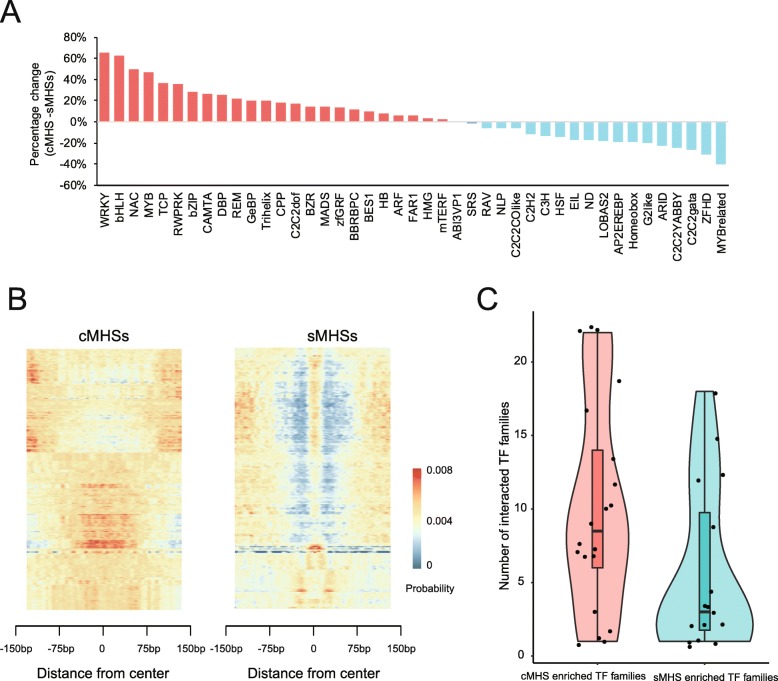
TF-binding patterns associated with cMHSs and sMHSs, respectively. **a** cMHSs and sMHSs associated with different TF families. Percentage of cMHSs and sMHSs associated with each TF family was calculated, and the percentage change for each TF family was calculated by minus the percentage of sMHSs from the percentage of cMHSs. Positive percentage change indicates that TF families are more enriched in cMHSs than in sMHSs. Negative percentage change indicates that TF families are more enriched in sMHSs than in cMHSs. **b** Positional bias of TF-binding motifs within cMHSs and sMHSs. The probabilities of motifs at each base pair within the ± 150-bp region from the center of sMHSs and cMHSs were calculated by CentriMo. The probabilities of TF-binding motifs at each base pair were plotted as heatmap and each line in the heatmap represents one motif. **c** Boxplot of numbers of TF families that interact with cMHSs- or sMHSs-enriched TF families

The distinct patterns of nucleosome organization, epigenetic modifications, and transcription of cognate genes associated with sMHSs and cMHSs inspired us to explore potential TFs that bind to these two different types of genomic regions. sMHS and cMHS sequences were searched for enrichment of known motifs that bind to a total of 529 TFs in *Arabidopsis* [41]. Interestingly, sMHSs and cMHSs were differentially enriched with motifs associated with distinct sets of TFs (Fig. 7**a**). For example, 65% of cMHSs contained motifs associated with the WRKY TFs, which are mostly involved in the regulation of pathogen responses and salicylic acid signaling [42, 43]. In contrast, only 8% sMHSs contained the same motifs. Conversely, 25% sMHSs contained the motif associated with CRC, a TF belongs to C2C2YABBY family and is involved in flower development [44], and cMHSs were not enriched with the same motif (Fig. 7a). cMHSs were especially overrepresented for motifs associated with WRKY, bHLH, NAC, RWP-RK, MYB, and bZIP TF families, which are involved in multiple processes during development, whereas sMHSs overrepresented for motifs associated with MYB-related, ZFHD, Homeobox, and AP2EREBP families, which are involved in stress response, floral development and circadian clock [45–48].

To further understand the function of sMHSs, we used AME [49] to identify motifs that were enriched in sMHSs using cMHSs as control sequences. Interestingly, five of the top 30 most enriched TF motifs within sMHSs were associated with transcription repression (Additional file 4: Table S3), including RBE (*p* value = 1.06e−30), STZ (*p* value = 6.70e−39), RHL41 (*p* value = 3.42e−28), AZF1 (*p* value = 3.68e−33), and AZF2 (*p* value = 1.36e−44). RBE is a putative zinc finger transcriptional repressor [50] and was reported to repress the expression of *AGAMOUS*, a key gene in flower development [51]. RBE binds to the promoter of microRNA gene *MIR164c* and represses its expression in flowers [52]. RHL41, also known as ZAT12, downregulates genes in CBF-mediated cold response pathway [53]. AZF1, AZF2, and STZ all belong to ZPT2-related proteins and act as transcriptional repressors in plant cells [54]. A recent study showed that AZF1 interacts with PRC2 complex and guides PRC2 complex to target regions to load H3K27me3 and repress gene expression [55].

### Organization of TF-binding motifs within sMHSs and cMHSs

A recent study in humans demonstrated that binding sites of different TFs show positional bias within the nucleosome-depleted regions (NDRs): some TFs tend to bind toward the edges within NDRs, some at the center, and some at other positions [56]. Giving the distinct sizes of cMHSs (262 bp) and sMHSs (85 bp), we wondered if the TF-binding motifs within cMHSs and sMHSs are organized differently. We analyzed the positions of all TF-binding sites by calculating the density of motifs within ± 150-bp regions flanking the center of sMHSs and cMHSs, respectively. The TF-binding sites generally distributed broadly within cMHSs. By contrast, the majority of the TF-binding sites concentrated at the center of sMHSs (Fig. 7b). We analyzed the positions of the motifs from the top five enriched TF families associated with cMHSs (WRKY, bHLH, NAC, MYB, and TCP) (Fig. 7a). These five TF families, including 89 TFs, showed wide distribution around cMHSs (Additional file 1: Figure S11A). cMHSs are longer in length than sMHSs and generally contain multiple TF binding sites, which may facilitate synergistic interaction among different TFs [57, 58]. Thus, we predict more TF-TF interactions for cMHS-enriched TF families than sMHS-enriched TF families. To test this hypothesis, we analyzed the number of TF families that interact with cMHS- and sMHS-enriched TF families using TF interaction data from BioGRID (The BioGRID interaction database: 2017 update). The cMHS-enriched TF families interact with more TF families than sMHS-enriched TF families (10 versus 6 TF families, *p* value = 0.047, *t*-test) (Fig. 7c and Additional file 1: Figure S11B).

## Discussion

MNase has been used in various types of chromatin studies. It was most commonly used to study nucleosome occupancy and positioning in eukaryotic genomes, which can be accomplished by sequencing mononucleosomal DNA fragments generated from MNase digestion [26]. Sequencing of DNAs derived from different levels of MNase digestion can assess the variability of chromatin accessibility in the entire genome [59–61]. Alternatively, sequencing of both nucleosomal and subnucleosomal DNA fragments derived from MNase digestion reveals the genomic positions of subnucleosome-sized particles associated with regulatory proteins [20, 22, 62]. Previous studies showed the potential of MNase to identify genomic regions associated with CREs. We modified the procedure of MNase-based chromatin assays by isolating and sequencing DNA fragments associated exclusively with subnucleosome-sized particles in Arabidopsis. This procedure allows enrichment of DNA fragments derived from genomic regions that are hypersensitive to MNase. We demonstrate that this procedure reveals open chromatin regions that were not identified by previous assays based on DNase I or Tn5.

Several previous studies showed that open chromatin regions identified by DNase I and Tn5 largely overlap in both animal and plant species [6, 63, 64]. We demonstrate that MH-seq reads cover the majority of the open chromatin regions identified DNase-seq and ATAC-seq. Remarkably, sMHSs, which represent 22% of the total MHSs, are not covered by DNase-seq or ATAC-seq reads. This could potentially be caused by different levels of sensitivity of the three enzymes to chromatin. MNase is not as readily as DNase I to cleave DNA sequences protected by nucleosomes [65]. MNase preferentially cleaves at linker sequences and trims the DNA sequences to core regions protected by nucleosomes [21]. In contrast, DNase I and Tn5 can cut within nucleosomes and generate a 10.5-bp periodicity of cutting pattern which represents translational phasing of nucleosomes [6, 66]. MNase cuts double-stranded DNA more efficiently at nucleotides A and T, but is less sensitive to polyA and polyT sequences [67]. DNase I is commonly considered as a nonspecific nuclease; however, preferred sites with A-T base-pairing and poor sites containing G-C base-pair have been reported [68]. Tn5 showed insertional biases toward sequences containing G and C [69]. These cutting/insertional biases of these three enzymes may cause the difference in revealing different classes of open chromatin.

**Fig. 8:**
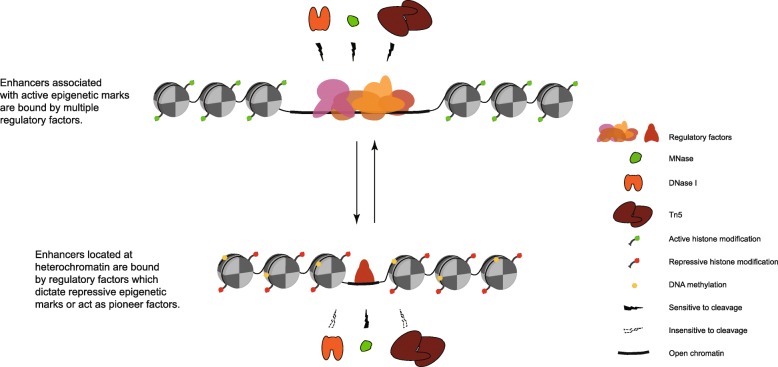
Diagram of enzyme digestion at regions associated with different classes of open chromatin. Regulatory proteins located in “repressive” open chromatin showed a relatively narrow open space and can only be accessed to MNase, but not to DNase I or Tn5

Alternatively, the sizes (molecular weight) of the three enzymes may be responsible for their differential accessibility to chromatin. MNase is a small protein with only 17 kDa [70]. By contrast, DNase I is 31 kDa [70] and Tn5 is 53 kDa [71]. Thus, the open space in some chromatin regions is likely sufficient for the access of MNase, but not DNase I or Tn5 (Fig. 8). This is supported by the fact that a high proportion of sMHSs is associated with H3K27me3 and DNA methylation, which are epigenetic marks typically associated with heterochromatin or more condensed chromatin. The DNase I and Tn5 sensitivity associated with sMHSs are significantly lower than cMHSs (Fig. 3a), which supports the notion that chromatin marked by sMHSs is less accessible to DNase I. In addition, open chromatin region marked by DHSs or cMHSs are flanked by phased nucleosomes in both rice [31] and Arabidopsis, but sMHSs are not flanked with well-positioned nucleosomes (Fig. 3b), which also supports the hypothesis on differential chromatin condensation associated with cMHS and sMHS domains.

Genes associated with sMHSs showed lower expression levels in leaves than in flower buds (Fig. 6b), suggesting that many of these genes are likely repressed in leaf tissue. There are several lines of evidence to support the enrichment of repressive factors associated with sMHSs. A significant proportion of sMHSs is associated with H3K27me3 and DNA methylation, both are well known repressive epigenetic marks associated with gene silencing [39, 72]. Some sMHSs show increased DNase I accessibility in flower tissue and their cognate genes are associated with active transcription in flowers (Fig. 6), which support the notion that these sMHSs were associated with repressive factors in leaves. We also identified several repressive sequence motifs enriched in sMHSs (Additional file 4: Table S3). Collectively, we demonstrate that MH-seq provides an important tool to identify and catalog all classes of open chromatin, especially those that are not accessible to DNase I and Tn5, which appear to be enriched with repressive factors and poised for function in different tissues.

## Conclusions

MNase, an enzyme with a small molecular weight, can be used to identify open chromatin regions that are not accessible to DNase I or Tn5. The open chromatin identified by MNase, but not DNase I or Tn5, shows distinct features associated with epigenetic marks H3K27me3 and DNA methylation. MH-seq provides an important tool to identify and catalog all classes of open chromatin in plants.

## Methods

### Library development for MH-seq

Sterilized seeds of *A. thaliana* ecotype Columbia (Col-0) were germinated in half-strength MS medium and grown under a 16-h light/8-h dark cycle at 23 °C for 2 weeks. Two-week-old seedling tissues were collected and immediately fixed with 1% of final concentration of formaldehyde under vacuum for 10 min at room temperature, then followed by 5 min neutralization by adding 0.125 M as the final concentration of glycine. Approximately 2.0 g of fixed seedling tissues was ground into a fine powder in liquid nitrogen and nuclei prepared following published protocols [73]. The final purified pellet of nuclei was suspended in 2.6 ml MNase digestion buffer (MNB, 10% sucrose, 50 mM Tris-HCl, pH 7.5, 4 mM MgCl_2_, and 1 mM CaCl_2_). The suspended nuclei were divided into 5 of 1.5-ml Eppendorf tubes (500 μl per aliquot) after aliquoting 100 μl of nuclei as an undigested control. The aliquoted nuclei were cleaved at 37 °C for 10 min using a series of MNase (N3755-200UN, Sigma) in 0 unit (U), 0.04 U, 0.2 U, 0.4 U, 0.8 U, and 1.2 U, respectively. Each MNase cleaved nuclei aliquot was split into two separate parts, one cross-linked part was stored at 4 °C overnight, and the second part was incubated at 65 °C overnight for reverse cross-linking. The cross-linked or reverse cross-linked nuclei were extracted by adding 1 volume of phenol:chloroform mixture. The recovered reverse cross-linked DNA was separated by running 2% of agarose gel in 1× TAE buffer. To exclude nucleosome-related DNA fragments, we specifically recovered DNA fragments with less than 100 bp (indicated by the red box in Additional file 1: Figure S1A) from 0.8 U of MNase-digested nuclei. The recovered small-sized DNA fragments were used for the construction of Illumina library following standard Illumina library preparation procedures (Additional file 1: Figure S1B). Each MH-seq dataset is associated with a different level of background, which is associated with the quality of the plant tissue and can be reduced by minimizing the degradation of the chromatin during library development.

### MH-seq data processing and peak calling

The MHS libraries were sequenced in single end mode to 100 nt on the Illumina Hiseq 2000 sequencing platform. Raw reads were trimmed using Cutadapt [74] and aligned to Arabidopsis genome (TAIR10, https://www.arabidopsis.org/) with Bowtie2 [25] using parameter “--local --very-sensitive-local”. Reads with mapping quality greater than 10 and full-length alignments were retained for further analysis.

We developed Jazz software (https://github.com/zhangtaolab/Jazz/) to identify MHSs. A MHS is defined as a genomic region that is significantly enriched with MH-seq reads compared to the genomic background. The threshold of the read count is determined using a cut-off value of the Bayes Factor (BF) of two competing hypotheses. A zero-inflated Poisson (ZIP) model is applied to determine the local threshold and identify peaks with each MHSs. This method can reduce the impact of excessive windows with zero read counts and reduce the false positives.

BEDTools [75] was used for the annotation of MHS peaks with different genomic features and to compare the DHSs, ATAC-seq peaks, and ChIP-seq peaks from Additional file 2: Table S1. The 22 TFs includes AGL15 [76], AP1 [77], AP2 [78], AP3 [79], BES1 [80], EIN3 [81], ERF115 [82], FHY3 [83], FLM [84], GL1 [85], GL3 [85], GTL1 [86], LFY [87] [88], PI [79], PIF3 [89], PIF4 [90], PIF5 [91], PRR5 [92], PRR7 [93], SEP3 [94], SOC1 [95, 96], and TOC1 [97]. The read coverage tracks were generated using bamCoverage from deepTools [98] with parameters “--centerReads –binSize 1. To plot the distribution of MHSs, chromosomes were divided into 10-kb non-overlapping windows and the number of MHSs calculated for each window. IGV [99] was used to display MHS peaks and other genomic tracks.

### Genomic and epigenomic features associated with MHSs

Open chromatin data (DNase-seq) [13] and ATAC-seq [15] and epigenomic data (H3K27me3 and H3K27ac) [28], nucleosome positioning (MNase-seq) [26], H2A.Z [100], and DNA methylation [101] were downloaded from NCBI (https://www.ncbi.nlm.nih.gov/). A complete list of the data used can be found in Additional file 5: Table S4. H3K27me3 and H3K27ac peaks were identified by SICER [102]. Clustering analyses were conducted using R package “kmeans”. Total within-clusters sum of squares (TWSS) of *k* from 2 to 16 were calculated. The optimal value of *k* (*k* = 5) was determined as the smallest value of *k* that the TWSS changed slower (Additional file 1: Figure S7C). The heatmaps of clusters were plotted using R package “pheatmap”. Profiles of the open chromatin data and epigenomic marks were generated by deeptools [98] and plotted using R.

Genes were assigned to MHSs if they are located within 1 kb up- and downstream of the MHS. If there are two genes on both sides of MHSs, the closest genes were selected. No directionality were required as enhancers can function in both directions. Gene expression data of leaves and flower buds were obtained from previous study of our group [13]. Briefly, RNA-seq data were trimmed by Cutadapt and aligned to Arabidopsis genome by Tophat [103]. Gene expression levels, measured as FPKM, were calculated using Cufflinks [104]. For comparing the gene expression changes between wild type and *ddm1*, RNA-seq data of wild type and *ddm1* were obtained from previous study [105]. Differentially expressed genes were identified using cuffdiff from cufflinks with *q* value < 0.05. Gene expression data of 79 tissues were obtained from TraVA [106] (http://travadb.org). Shannon entropy of each gene was calculated using R package “entropy”. GO enrichment analysis were conducted using PANTHER [107] provided by TAIR10 (https://www.arabidopsis.org).

Whole genome bisulfite sequencing data from wild type [101] and *ddm1* [38] were mapped to reference genome using bismark [108]. Bismark_methylation_extractor from bismark software was used to extract methylation information of each cytosine. A cytosine is methylated to have more 60% CG methylation, 20% CHG methylation, and 5% CHH methylation. The methylated regions were then identified using a slide-window-based method. The average methylation level in each window which contains five continuous CG, CHG, or CHH was calculated and window slid in a step of one CG, CHG, or CHH. Windows with less than four methylated cytosines were discarded. CG, CHG, and CHH methylated regions were merged to form union sets of methylated regions.

### Motif analysis and functional validation of MHSs

The candidate TF-binding sites were detected using Find Individual Motif Occurences (FIMO) [109] against the Arabidopsis cistrome database [41] (http://neomorph.salk.edu/PlantCistromeDB) with default parameters. Differentially enriched motifs were detected using Motif Enrichment Analysis (AME) [49] with default parameters. The preferential positioning analysis was conducted using centrimo with “--local” and defaults for other parameters.

The enhancer validation was conducted using a previously described method [35]. The DNA sequences of MHSs were synthesized and cloned into vector pCAMBIA-CRE-LUC, which contains a firefly luciferase reporter gene and the minimal cauliflower mosaic virus (CaMV) 35S promoter (− 50 to − 2 bp). The MHSs were placed upstream of the mini35S promoter. The constructs were then infiltrated into leaves of 14-day-old *N. benthamiana* mediated by agrobacterium. The bioluminescent signals were detected by NightSHADE LB 985 (Berthold Technologies, USA).

## Supplementary information

Additional file 1: **Figure S1.** DNA fragments recovered for MH-seq. **Figure S2.** Genomic locations of all MHSs and overlap between TF-binding sites and MHSs. **Figure S3.** Positions of nucleosomes, MNase and DNase I cuts on MHSs. **Figure S4.** Examples of cMHSs and sMHSs. **Figure S5.** Distribution of MHSs along chromosome 1 of *A. thaliana*. **Figure S6.** Histone modifications and DNA methylations associated with sMHSs and cMHSs. **Figure S7.** Clustering analysis of all MHSs based on epigenetic marks H3K27ac, H2A.Z, H3K27me3 and DNA methylation (CG, CHG and CHH). **Figure S8.** Expression patterns of genes associated with sMHSs [110]. **Figure S9.** CG, CHG and CHH DNA methylation profile at genes in class 2. **Figure S10.** Changes of DNA methylation and gene expression levels of class 2 in *ddm1*. **Figure S11.** Positioning of TF-binding motifs within cMHSs and correlation between number of TF families and percentage change. (PDF 10155 kb)

Additional file 2: **Table S1.** TFs and their genome-wide binding sites in *A. thaliana. (DOCX 21 kb)*

Additional file 3: **Table S2.** sMHSs selected for functional validation based on agrobacterium-mediated transient assay. (XLSX 14 kb)

Additional file 4: **Table S3.** Top 30 most enriched TFs and their binding motifs within sMHSs. (XLSX 14 kb)

Additional file 5: **Table S4.** List of data sets used in current study. (DOCX 34 kb)

Additional file 6. Review history (DOCX 278 kb)
