# Supplementary material for: Genome-wide MNase hypersensitivity assay unveils distinct classes of open chromatin associated with H3K27me3 and DNA methylation in Arabidopsis thaliana
Source: Genome Biol. 2020 Feb 3;21:24. doi: 10.1186/s13059-020-1927-5 (PMC6996174; doi:10.1186/s13059-020-1927-5)
Supplement: Supplementary file 1 — Figure S1. DNA fragments recovered for MH-seq. Figure S2. Genomic locations of all MHSs and overlap between TF-binding sites and MHSs. Figure S3. Positions of nucleosomes, MNase and DNase I cuts on MHSs. Figure S4. Examples of cMHSs and sMHSs. Figure S5. Distribution of MHSs along chromosome 1 of A. thaliana. Figure S6. Histone modifications and DNA methylations associated with sMHSs and cMHSs. Figure S7. Clustering analysis of all MHSs based on epigenetic marks H3K27ac, H2A.Z, H3K27me3 and DNA methylation (CG, CHG and CHH). Figure S8. Expression patterns of genes associated with sMHSs [110]. Figure S9. CG, CHG and CHH DNA methylation profile at genes in class 2. Figure S10. Changes of DNA methylation and gene expression levels of class 2 in ddm1. Figure S11. Positioning of TF-binding motifs within cMHSs and correlation between number of TF families and percentage change. (PDF 10155 kb) [file 13059_2020_1927_MOESM1_ESM.pdf]

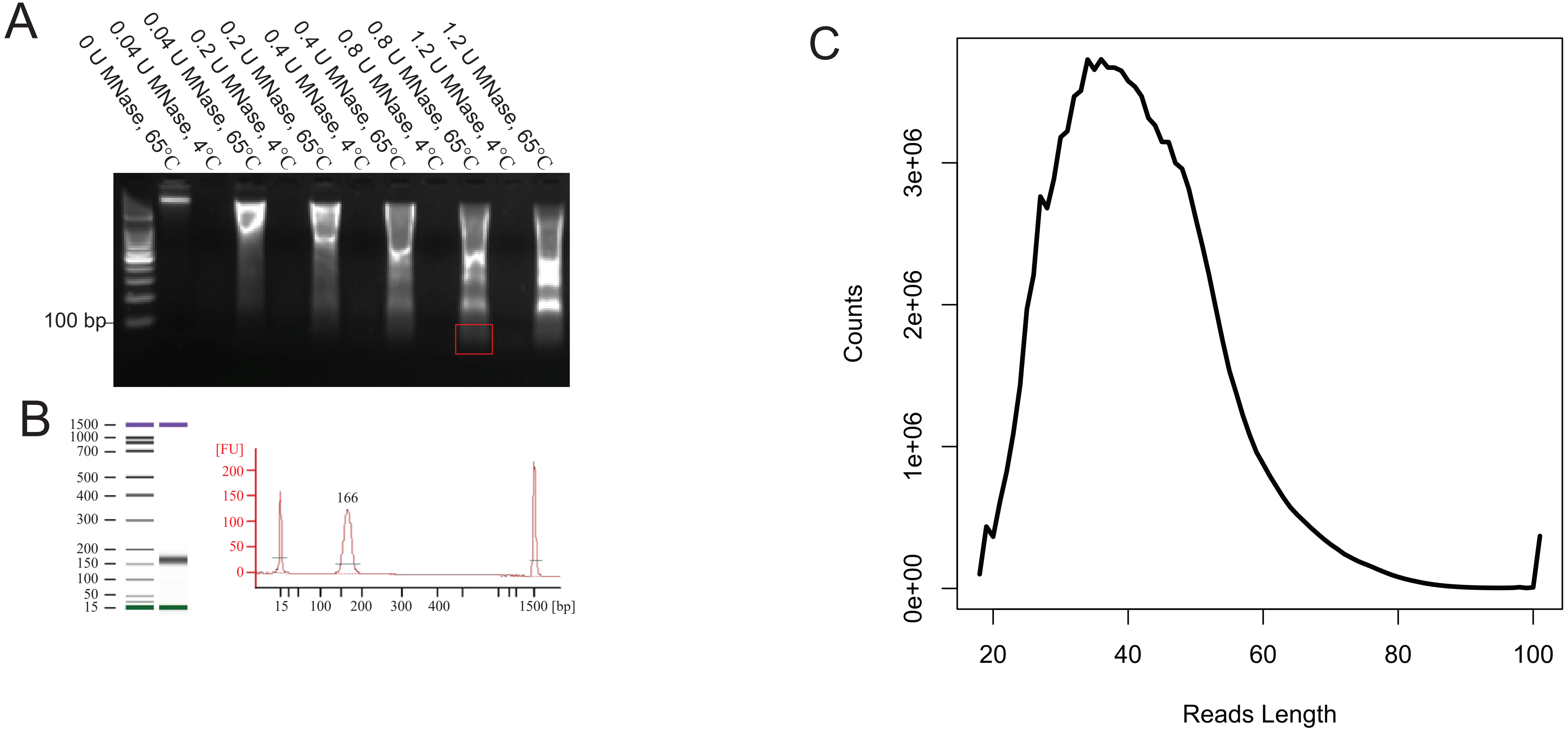

Figure S1. DNA fragments recovered for MH-seq. (**A**) Separation of MNase-digested DNA in 2% of agarose gel. Nuclei were isolated from Arabidopsis seedling tissues, cross-linked by formaldehyde, and digested with different concentrations of MNase as indicated in the figure. MNase-digested DNA was prepared from either cross-linked nuclei (MNased fixed nuclei kept at 4°C overnight) or reverse cross-linked nuclei (MNased fixed nuclei incubated at 65°C overnight) using phenol based extraction method, and was separated by running 2% agarose gel. The DNA fragments with size less than 100 bp indicated by red box were recovered from reverse cross-linked nuclei after cleavage with 0.8 U MNase for library construction. (**B**) Analysis of the library DNA on Bioanalyzer 2100. Left panel: gel virtual view of library DNA; Right panel: electropherogram view of the library DNA peak, the observed average size of the library is 166 bp. (**C**) Read length distribution of single-end sequencing data from the library.

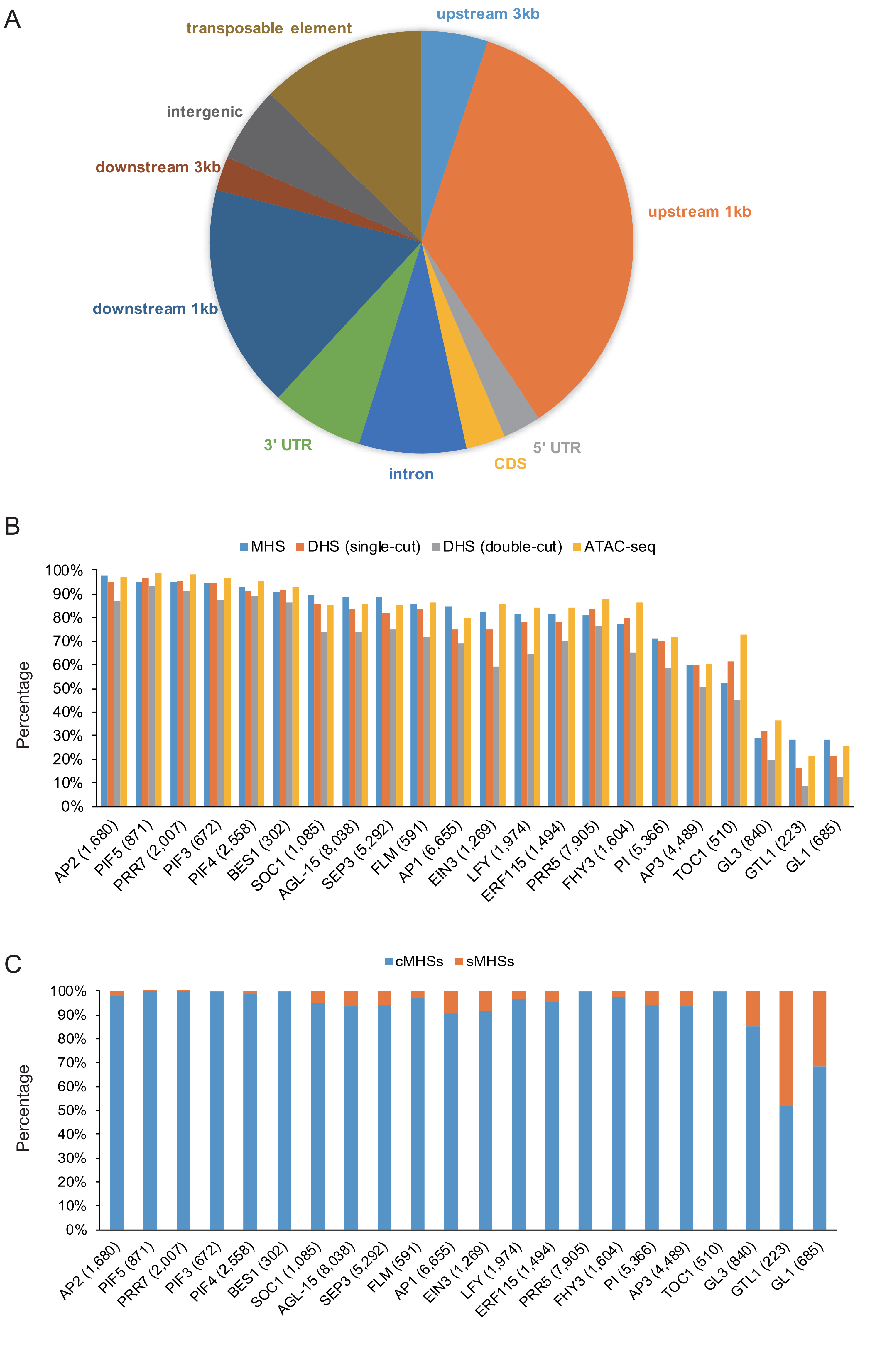

Figure S2. Genomic locations of all MHSs and overlap between TF-binding sites and MHSs. (**A**) Genomic locations of all MHSs. (**B**) Overlap between TF-binding sites and open chromatin identified by MH-seq, DNase-seq and ATAC-seq. *y*-axis represents the percentages of TF-binding sites that overlaps with open chromatin regions identified by the four techniques. *x*-axis shows the list of TFs. Number of TF-binding sites is listed in the parentheses after each TF. (**C**) Overlap between TF-binding sites and cMHSs and sMHSs. *y*-axis represents the percentages of TF-binding sites that overlaps with cMHSs and sMHSs. *x*-axis shows the list of TFs. Number of TF-binding sites is listed in the parentheses after each TF.

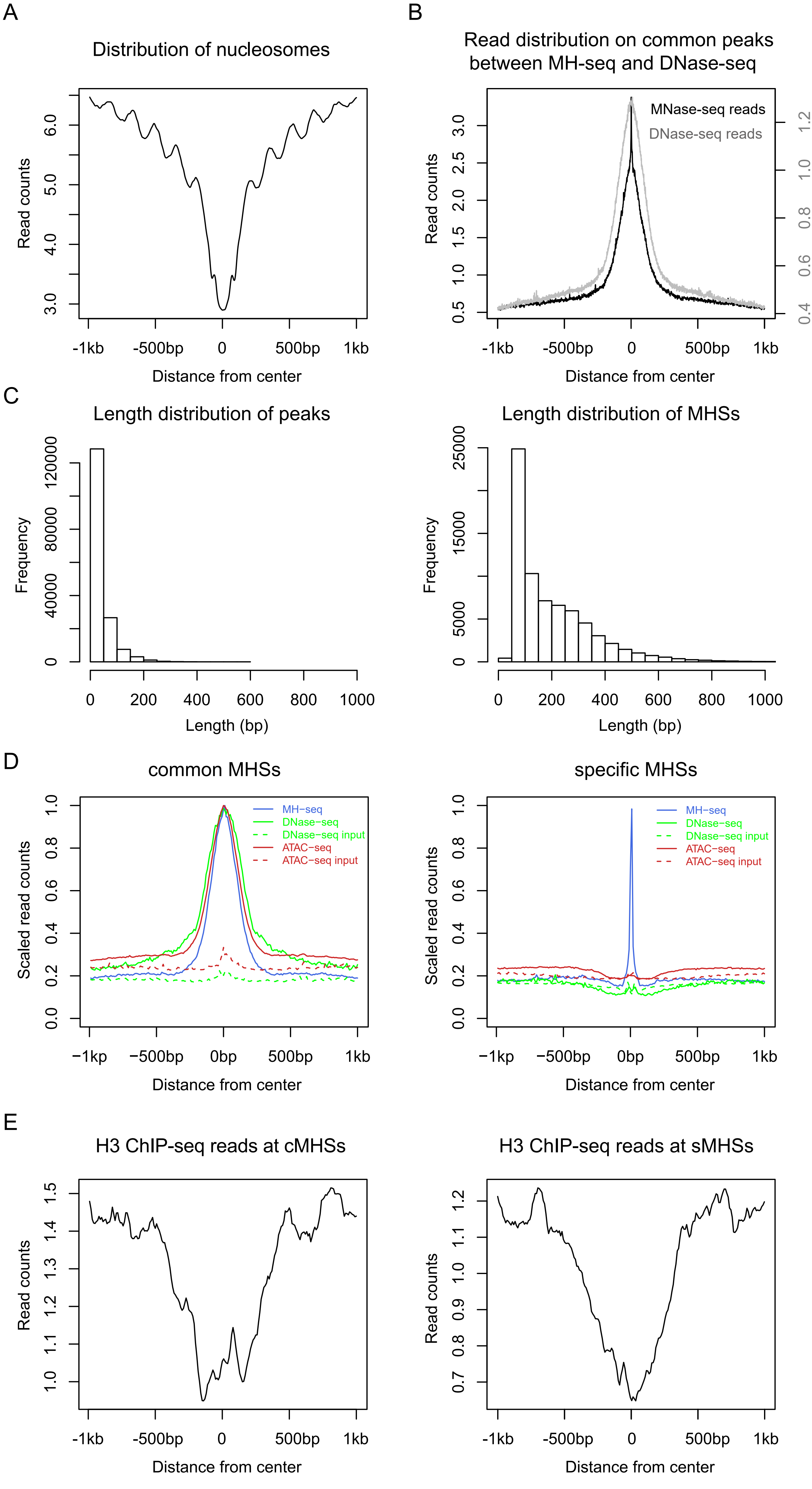

Figure S3. Positions of nucleosomes, MNase and DNase I cuts on MHSs. **(A)** Distribution of nucleosome at MHSs. MHSs were aligned at the midpoints and average number of MH-seq reads at each base pair spanning ±1 kb from the midpoint was plotted. **(B)** MHSs were aligned at the midpoints and average number of MH-seq and DNase-seq reads at each base pair spanning ±1 kb from the midpoint was plotted. **(C)** Length distribution of MHSs and peaks within MHSs. **(D)** Distribution of MH-seq, DNase-seq, ATAC-seq, DNase-seq input and ATAC-seq input reads at common MHSs and specific MHSs. The inputs of DNase-seq and ATAC-seq were generated from DNase I and Tn5 digested naked DNA, respectively. **(E)** Distribution of H3 ChIP-seq reads at common MHSs and specific MHSs.

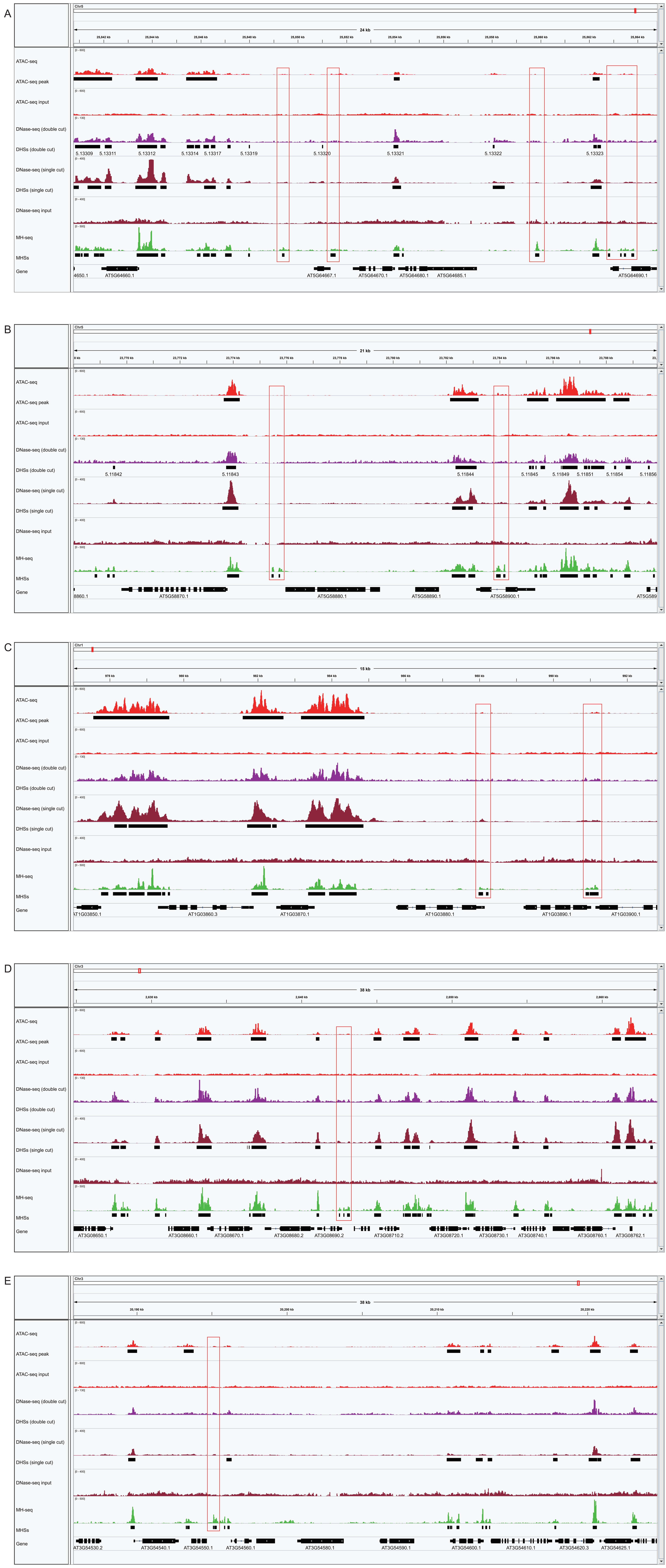

Figure S4. Examples of cMHSs and sMHSs. Profiles from ATAC-seq and DNase-seq data (single-cut and double-cut) are also included for comparison. Open chromatin regions are marked by black rectangle under profile of each technique. sMHSs were marked by red rectangles. Gene models are shown at the bottom.

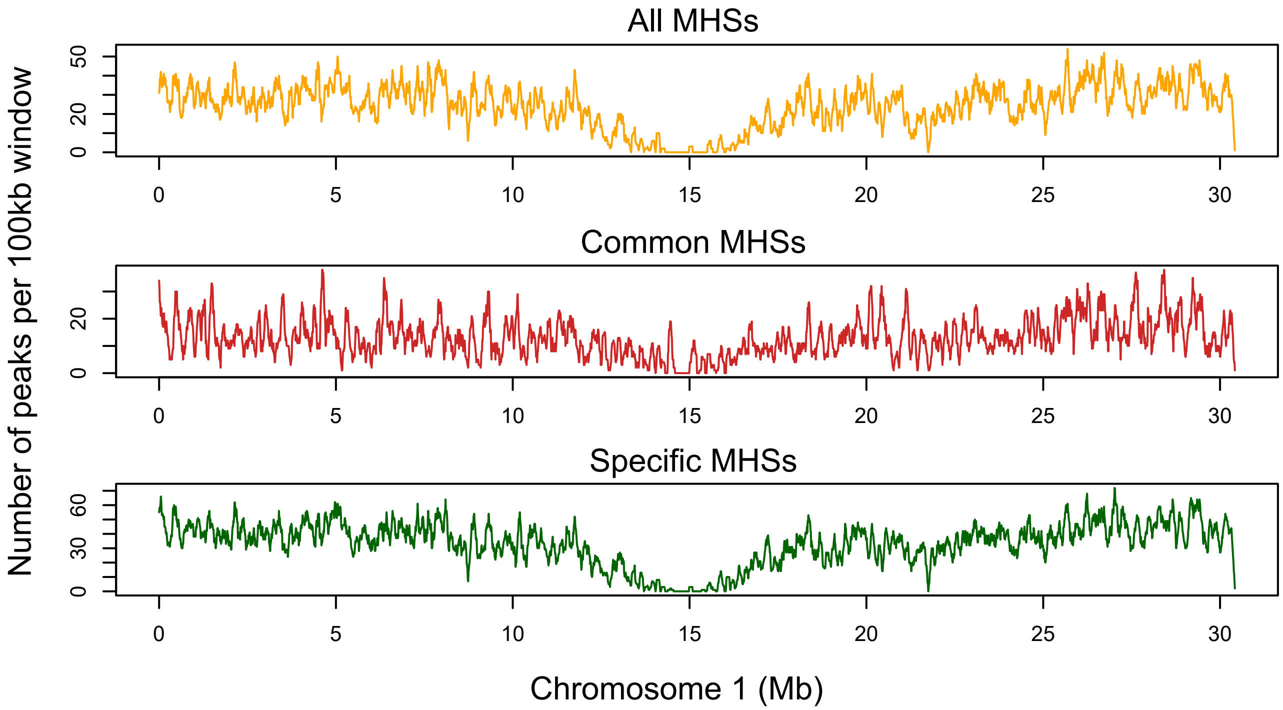

Figure S5. Distribution of MHSs along chromosome 1 of *A. thaliana*. The sequence of chromosome 1 was divided into 10-kb non-overlapping windows. The number of MHSs within each window was calculated and plotted.

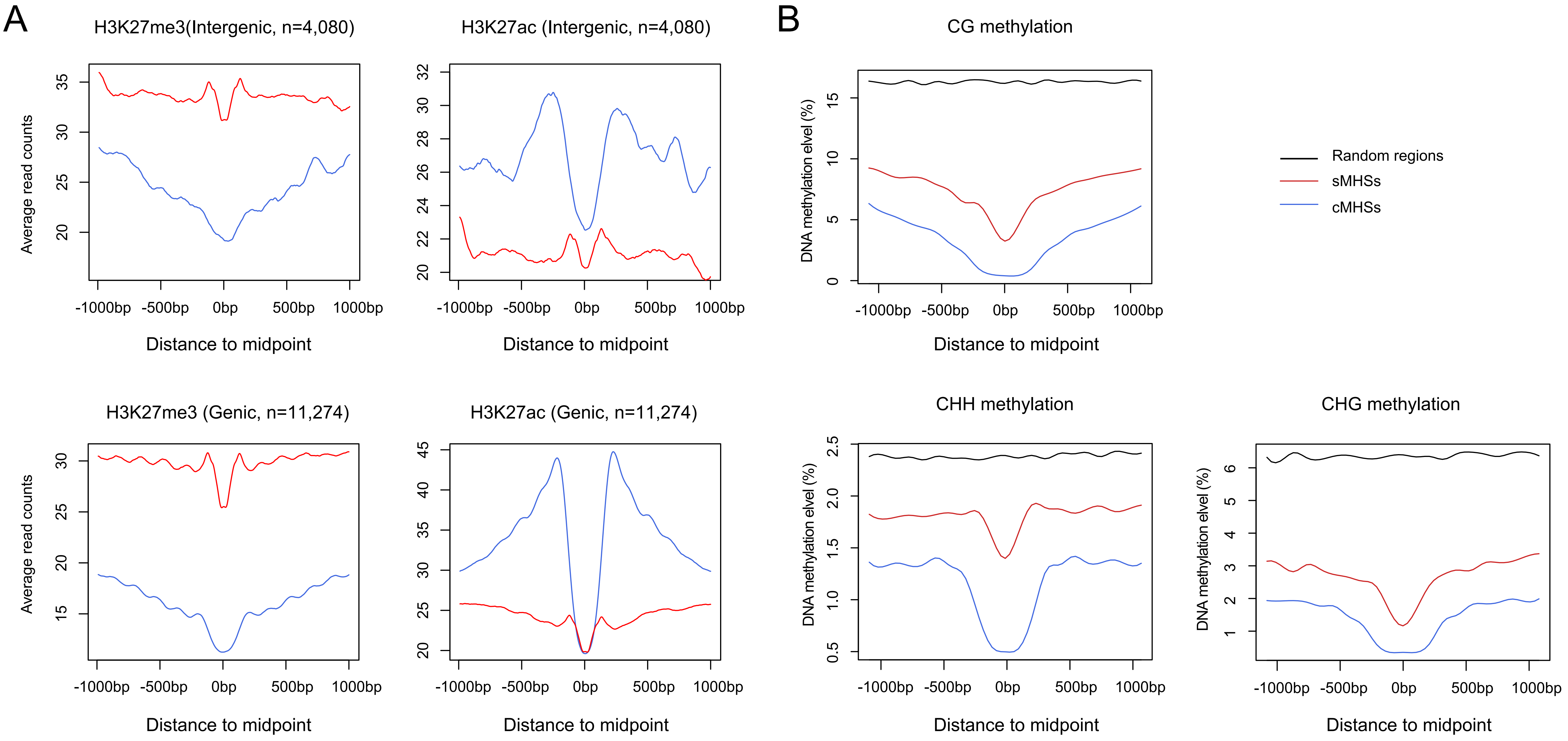

Figure S6. Histone modifications and DNA methylations associated with sMHSs and cMHSs. **(A)** Distribution of H3K27me3 and H3K27ac at sMHSs (red line) and cMHSs (blue line). A genic MHS is located within 1 kb upstream or downstream of a gene. An intergenic MHS is located more than 1 kb upstream and downstream of a gene. **(B)** DNA methylation of sMHSs and cMHSs. CG, CHG and CHH methylation levels of sMHSs, cMHSs and genomic regions randomly selected from the genome were calculated and methylation levels were aggregated to make the plots.

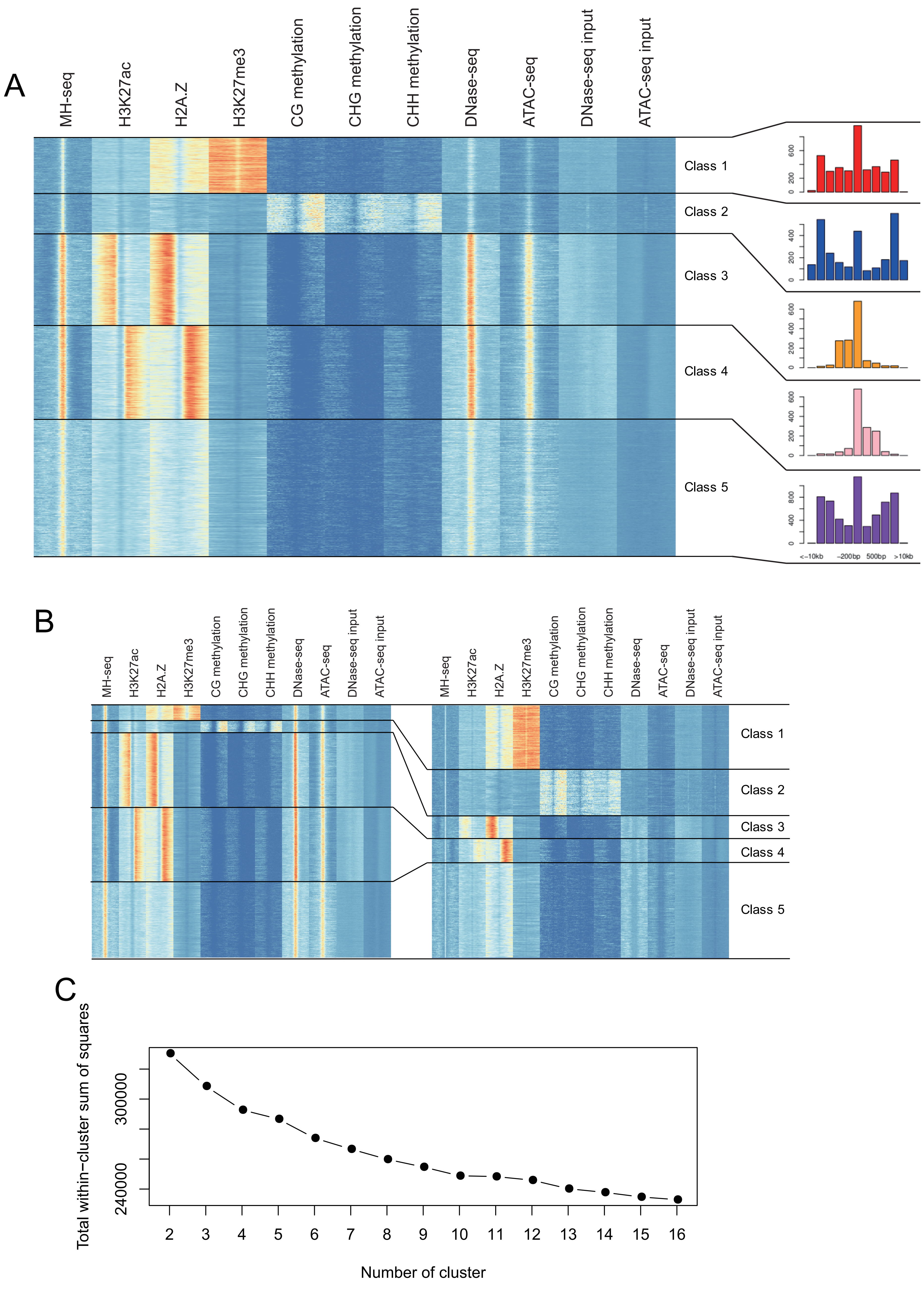

Figure S7. Clustering analysis of all MHSs based on epigenetic marks H3K27ac, H2A.Z, H3K27me3 and DNA methylation (CG, CHG and CHH). **(A)** Heatmap of clusters of all MHSs generated by k-means cluster analysis. Each window represents signals ±1 kb from the midpoints of cMHSs and sMHSs. Classes identified by k-mean cluster analysis were marked by black lines. The color key is the same as Figure 4. The right panel shows the distance between MHSs to closest genes. *y*-axis represents number of MHSs. *x*-axis represents the distance between MHSs to closest genes. Negative value means genes located at upstream of MHSs, positive value means genes located at downstream of MHSs. **(B)** Heatmap of clusters of sMHSs and cMHSs. The sMHSs and cMHSs from **(A)** were extracted and ploted separately. The color key is the same as Figure 4. **(C)** Elbow plot of k-mean clusters from k=2 to k=16. *x*-axis represents the number of clusters k. *y*-axis represents the total within-cluster sum of squares.

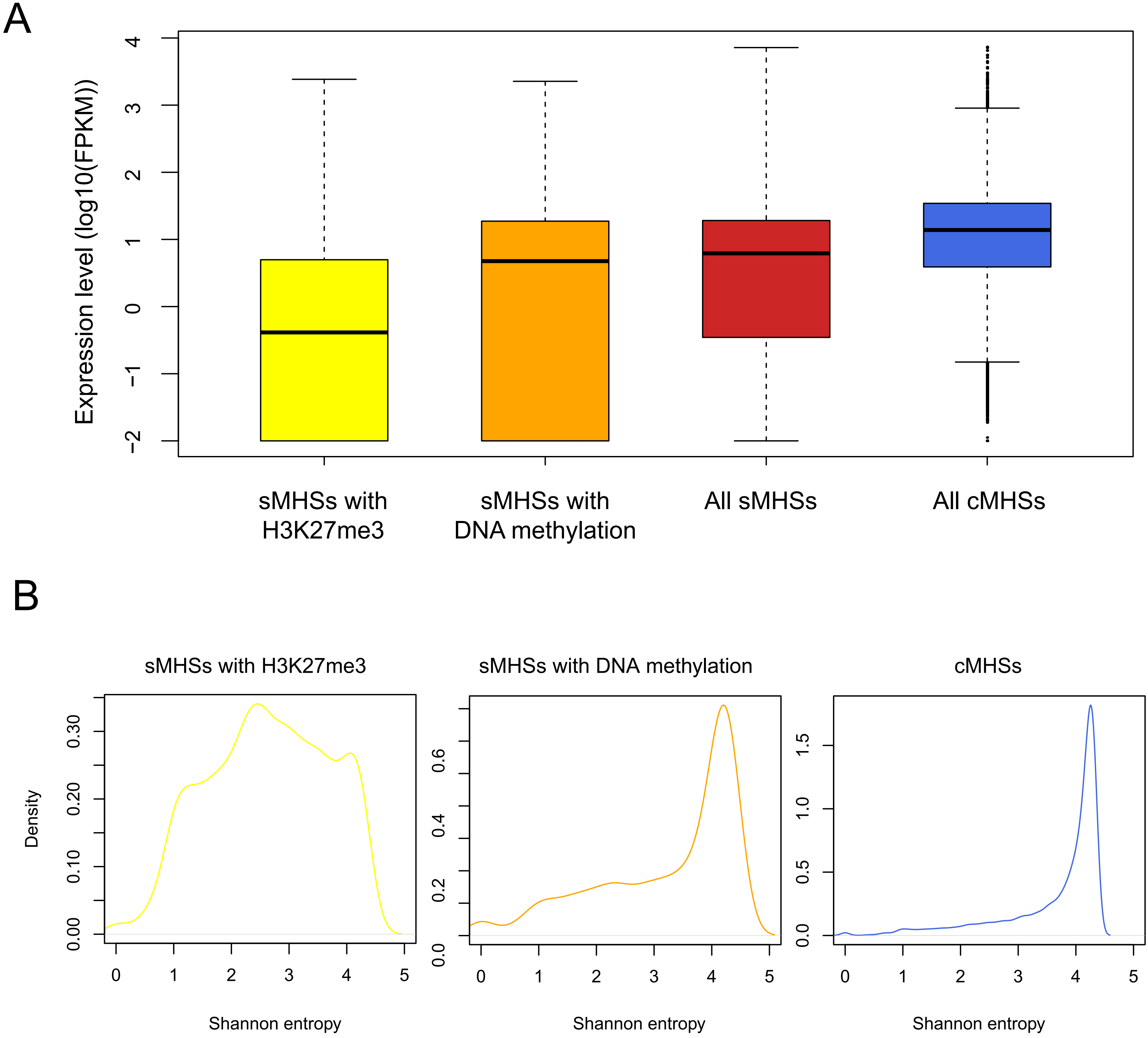

Figure S8. Expression patterns of genes associated with sMHSs. **(A)** Boxplots of expression levels of genes associated with different types of sMHSs. **(B)** Tissue specificity of genes associated with different types of sMHSs. *x*-axis represents Shannon entropy [110] calculated based on gene expression data from 79 tissues. *y*-axis represents the density of genes that have the Shannon entropy indicated on the *x*-axis.

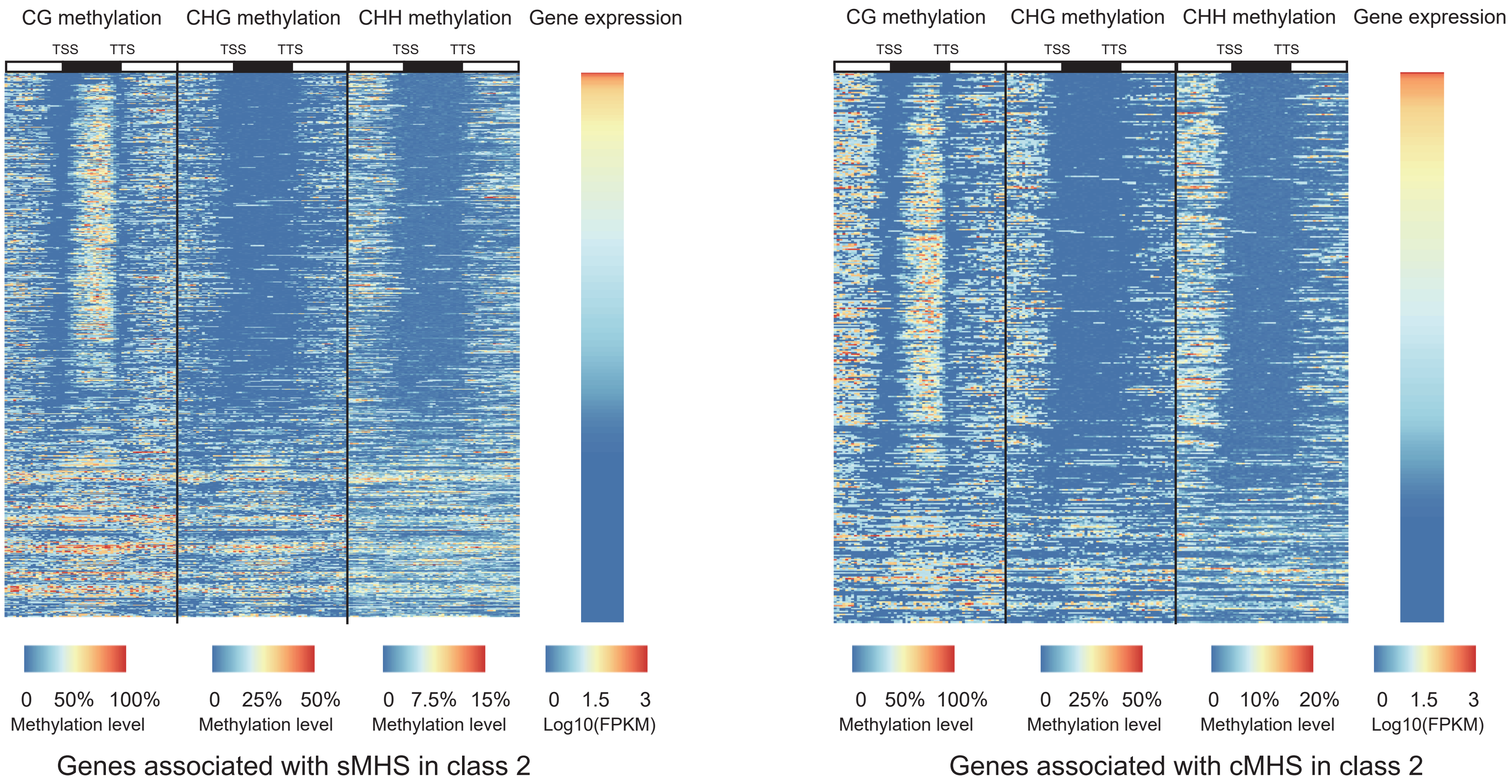

Figure S9. CG, CHG and CHH DNA methylation profile at genes in class 2. Gene bodies, 1kb upstream and 1kb downstream of genes were divided into 20 bins. The CG, CHG and CHH methylation levels in each bin were calculated and plotted as heatmap. Genes were sorted by expression level in the heatmap.

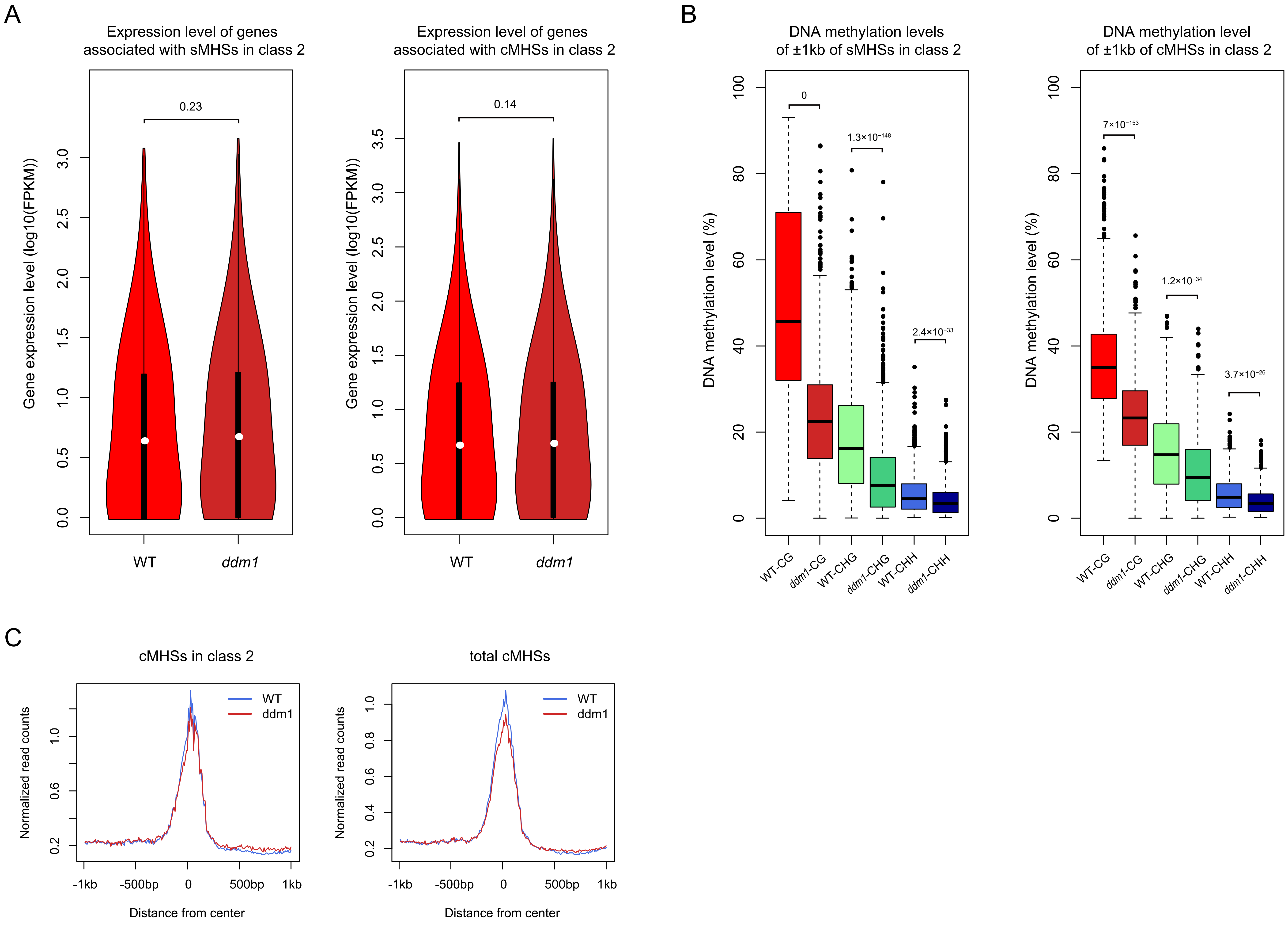

Figure S10. Changes of DNA methylation and gene expression levels of class 2 in *ddm1*. **(A)** Expression levels of genes in class 2 in wild type and *ddm1* mutant. **(B)** DNA methylation levels of MHSs in class 2 in wild type and *ddm1* mutant. The CG, CHG and CHH methylation levels of ±1kb of sMHSs and cMHSs were calculated and plotted as boxplot. Significance of difference between wild type and *ddm1* mutant was tested using Mann-Whitney' test. *P*-values were added in each pair of comparison. **(C)** DNase I sensitivity of cMHSs in class 2 in wild type and *ddm1* mutant. DNase I sensitivity of total cMHSs were plotted for comparison.

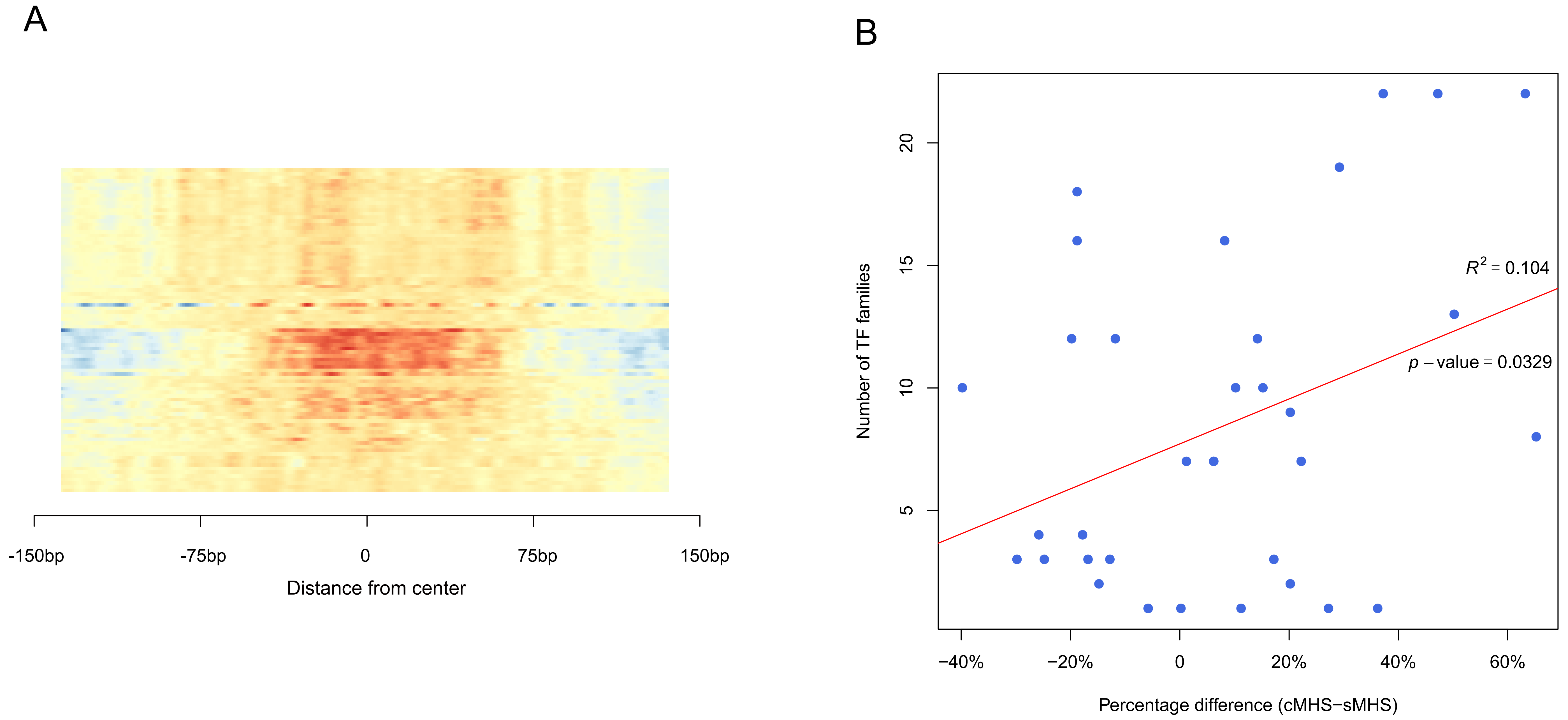

Figure S11. Positioning of TF-binding motifs within cMHSs and correlation between number of TF families and percentage change. **(A)** Positioning of motifs of the top five enriched TFs within cMHSs. **(B)** Correlation between percentage change and number of interacted TF families. *x*-axis represents percentage change of TFs as used in Figure 9A. *y*-axis represents number of interacted TF families with TF families that show a specific percentage change indicated on *x*-axis.
