# Supplementary material for: Genome-wide MNase hypersensitivity assay unveils distinct classes of open chromatin associated with H3K27me3 and DNA methylation in Arabidopsis thaliana
Source: Genome Biol. 2020 Feb 3;21:24. doi: 10.1186/s13059-020-1927-5 (PMC6996174; doi:10.1186/s13059-020-1927-5)
Supplement: Supplementary file 2 — Table S1. TFs and their genome-wide binding sites in A. thaliana. (DOCX 21 kb) [file 13059_2020_1927_MOESM2_ESM.docx]

**Table S1.** TFs and their genome-wide binding sites in *A. thaliana.*

| **TF** | **TF-binding sites** | **Tissue type** | **Reference** |
| --- | --- | --- | --- |
| AGL15 | 8,038 | Embryonic culture | (Zheng et al., 2009) |
| AP1 | 6,655 | 4-Week-old inflorescences | (Kaufmann et al., 2010) |
| AP2 | 1,680 | Young inflorescences | (Yant et al., 2010) |
| AP3 | 4,489 | Stage 5 floral buds | (Wuest et al., 2012) |
| BES1 | 302 | 14-d-old seedlings | (Yu et al., 2011) |
| EIN3 | 1,269 | 3-d-old seedlings | (Chang et al., 2013) |
| ERF115 | 1,494 | Cell culture | (Heyman et al., 2013) |
| FHY3 | 1,604 | 4-d-old seedling | (Ouyang et al., 2011) |
| FLM | 591 | 15-d-old seedlings | (Pose et al., 2013) |
| GL1 | 685 | 3-Week-old green tissue | (Morohashi and Grotewold, 2009) |
| GL3 | 840 | 3-Week-old green tissue | (Morohashi and Grotewold, 2009) |
| GTL1 | 223 | 2-Week-old whole aerial tissues | (Breuer et al., 2012) |
| LFY | 1,974 | 9-d-old seedlings | (Moyroud et al., 2011; Winter et al., 2011) |
| PI | 5,366 | Stage 5 floral buds | (Wuest et al., 2012) |
| PIF3 | 672 | 2-d-old seedlings | (Zhang et al., 2013) |
| PIF4 | 2,558 | 14-d-old seedlings | (Oh et al., 2012) |
| PIF5 | 871 | 10-d-old seedlings | (Hornitschek et al., 2012) |
| PRR5 | 7,905 | Whole plants | (Nakamichi et al., 2012) |
| PRR7 | 2,007 | 14-d-old seedlings | (Liu et al., 2013) |
| SEP3 | 5,292 | 5-Week-old inflorescences | (Kaufmann et al., 2009) |
| SOC1 | 1,085 | 15-d-old shoot apices | (Immink et al., 2012; Tao et al., 2012) |
| TOC1 | 510 | 14-d-old seedlings | (Huang et al., 2012) |

**Reference**

Breuer, C., Morohashi, K., Kawamura, A., Takahashi, N., Ishida, T., Umeda, M., Grotewold, E., and Sugimoto, K. (2012). Transcriptional repression of the APC/C activator CCS52A1 promotes active termination of cell growth. EMBO J 31, 4488-4501.

Chang, K.N., Zhong, S., Weirauch, M.T., Hon, G., Pelizzola, M., Li, H., Huang, S.S.C., Schmitz, R.J., Urich, M.A., Kuo, D., Nery, J.R., Qiao, H., Yang, A., Jamali, A., Chen, H.M., Ideker, T., Ren, B., Bar-Joseph, Z., Hughes, T.R., and Ecker, J.R. (2013). Temporal transcriptional response to ethylene gas drives growth hormone cross-regulation in Arabidopsis. Elife 2, e00675.

Heyman, J., Cools, T., Vandenbussche, F., Heyndrickx, K.S., Van Leene, J., Vercauteren, I., Vanderauwera, S., Vandepoele, K., De Jaeger, G., Van der Straeten, D., and De Veylder, L. (2013). ERF115 Controls Root Quiescent Center Cell Division and Stem Cell Replenishment. Science 342, 860-863.

Hornitschek, P., Kohnen, M.V., Lorrain, S., Rougemont, J., Ljung, K., Lopez-Vidriero, I., Franco-Zorrilla, J.M., Solano, R., Trevisan, M., Pradervand, S., Xenarios, I., and Fankhauser, C. (2012). Phytochrome interacting factors 4 and 5 control seedling growth in changing light conditions by directly controlling auxin signaling. The Plant journal : for cell and molecular biology 71, 699-711.

Huang, W., Perez-Garcia, P., Pokhilko, A., Millar, A.J., Antoshechkin, I., Riechmann, J.L., and Mas, P. (2012). Mapping the core of the Arabidopsis circadian clock defines the network structure of the oscillator. Science 336, 75-79.

Immink, R.G.H., Pose, D., Ferrario, S., Ott, F., Kaufmann, K., Valentim, F.L., de Folter, S., van der Wal, F., van Dijk, A.D.J., Schmid, M., and Angenent, G.C. (2012). Characterization of SOC1's central role in flowering by the identification of its upstream and downstream regulators. Plant Physiol 160, 433-449.

Kaufmann, K., Muino, J.M., Jauregui, R., Airoldi, C.A., Smaczniak, C., Krajewski, P., and Angenent, G.C. (2009). Target genes of the MADS transcription factor SEPALLATA3: integration of developmental and hormonal pathways in the Arabidopsis flower. Plos Biol 7, 854-875.

Kaufmann, K., Wellmer, F., Muino, J.M., Ferrier, T., Wuest, S.E., Kumar, V., Serrano-Mislata, A., Madueno, F., Krajewski, P., Meyerowitz, E.M., Angenent, G.C., and Riechmann, J.L. (2010). Orchestration of floral initiation by APETALA1. Science 328, 85-89.

Liu, T., Carlsson, J., Takeuchi, T., Newton, L., and Farre, E.M. (2013). Direct regulation of abiotic responses by the Arabidopsis circadian clock component PRR7. Plant J 76, 101-114.

Morohashi, K., and Grotewold, E. (2009). A systems approach reveals regulatory circuitry for Arabidopsis trichome initiation by the GL3 and GL1 selectors. Plos Genet 5, e1000396.

Moyroud, E., Minguet, E.G., Ott, F., Yant, L., Pose, D., Monniaux, M., Blanchet, S., Bastien, O., Thevenon, E., Weigel, D., Schmid, M., and Parcy, F. (2011). Prediction of regulatory interactions from genome sequences using a biophysical model for the Arabidopsis LEAFY transcription factor. Plant Cell 23, 1293-1306.

Nakamichi, N., Kiba, T., Kamioka, M., Suzuki, T., Yamashino, T., Higashiyama, T., Sakakibara, H., and Mizuno, T. (2012). Transcriptional repressor PRR5 directly regulates clock-output pathways. P Natl Acad Sci USA 109, 17123-17128.

Oh, E., Zhu, J.Y., and Wang, Z.Y. (2012). Interaction between BZR1 and PIF4 integrates brassinosteroid and environmental responses. Nature cell biology 14, 802-809.

Ouyang, X., Li, J., Li, G., Li, B., Chen, B., Shen, H., Huang, X., Mo, X., Wan, X., Lin, R., Li, S., Wang, H., and Deng, X.W. (2011). Genome-wide binding site analysis of FAR-RED ELONGATED HYPOCOTYL3 reveals its novel function in Arabidopsis development. Plant Cell 23, 2514-2535.

Pose, D., Verhage, L., Ott, F., Yant, L., Mathieu, J., Angenent, G.C., Immink, R.G.H., and Schmid, M. (2013). Temperature-dependent regulation of flowering by antagonistic FLM variants. Nature 503, 414-+.

Tao, Z., Shen, L., Liu, C., Liu, L., Yan, Y., and Yu, H. (2012). Genome-wide identification of SOC1 and SVP targets during the floral transition in Arabidopsis. Plant J 70, 549-561.

Winter, C.M., Austin, R.S., Blanvillain-Baufumé, S., Reback, M.A., Monniaux, M., Wu, M.-F., Sang, Y., Yamaguchi, A., Yamaguchi, N., Parker, J.E., Parcy, F., Jensen, S.T., Li, H., and Wagner, D. (2011). LEAFY target genes reveal floral regulatory logic, cis motifs, and a link to biotic stimulus response. Dev Cell 20, 430-443.

Wuest, S.E., O'Maoileidigh, D.S., Rae, L., Kwasniewska, K., Raganelli, A., Hanczaryk, K., Lohan, A.J., Loftus, B., Graciet, E., and Wellmer, F. (2012). Molecular basis for the specification of floral organs by APETALA3 and PISTILLATA. Proc Natl Acad Sci USA 109, 13452-13457.

Yant, L., Mathieu, J., Dinh, T.T., Ott, F., Lanz, C., Wollmann, H., Chen, X.M., and Schmid, M. (2010). Orchestration of the floral transition and floral development in Arabidopsis by the bifunctional transcription factor APETALA2. Plant Cell 22, 2156-2170.

Yu, X.F., Li, L., Zola, J., Aluru, M., Ye, H.X., Foudree, A., Guo, H.Q., Anderson, S., Aluru, S., Liu, P., Rodermel, S., and Yin, Y.H. (2011). A brassinosteroid transcriptional network revealed by genome-wide identification of BESI target genes in Arabidopsis thaliana. Plant J 65, 634-646.

Zhang, Y., Mayba, O., Pfeiffer, A., Shi, H., Tepperman, J.M., Speed, T.P., and Quail, P.H. (2013). A quartet of PIF bHLH factors provides a transcriptionally centered signaling hub that regulates seedling morphogenesis through differential expression-patterning of shared target genes in Arabidopsis. Plos Genet 9, e1003244.

Zheng, Y.M., Ren, N., Wang, H., Stromberg, A.J., and Perry, S.E. (2009). Global identification of targets of the Arabidopsis MADS domain protein AGAMOUS-Like15. Plant Cell 21, 2563-2577.
