# Supplementary material for: Genome-wide MNase hypersensitivity assay unveils distinct classes of open chromatin associated with H3K27me3 and DNA methylation in Arabidopsis thaliana
Source: Genome Biol. 2020 Feb 3;21:24. doi: 10.1186/s13059-020-1927-5 (PMC6996174; doi:10.1186/s13059-020-1927-5)
Supplement: Supplementary file 5 — Table S4. List of data sets used in current study. (DOCX 34 kb) [file 13059_2020_1927_MOESM5_ESM.docx]

**Table S4.** List of data used in current study

| **Data** | **Reference** | **NCBI repository** |
| --- | --- | --- |
| DNase-seq reads and DHSs | [1] | Gene Expression Omnibus GSE34318 |
| ATAC-seq reads and peaks | [2] | Gene Expression Omnibus GSE85203 |
| H3K27me3 ChIP-seq | [3] | Short Read Archive PRJNA252965 |
| H3K27ac ChIP-seq | [3] | Short Read Archive PRJNA252965 |
| Nucleosome MNase-seq | [4] | Short Read Archive SRR1536110 |
| H2A.Z ChIP-seq | [5] | Short Read Archive SRR5364422 |
| Wild type DNA methylation | [6] | Short Read Archive SRR2392658 |
| *ddm1* DNA methylation | [7] | Short Read Archive SRR771518 |
| DNase-seq input | Not available | Short Read Archive SRR10051102 |
| ATAC-seq input | [8] | Short Read Archive SRR5829244 |
| RNA-seq of Col and *ddm1* | [9] | Short Read Archive SRR2878548, SRR2878549, SRR2878550, SRR2878560, SRR2878561 and SRR2878562 |

**References**

1. Zhang WL, Zhang T, Wu YF, Jiang JM: **Genome-wide identification of regulatory DNA elements and protein-binding footprints using signatures of open chromatin in Arabidopsis.** *Plant Cell* 2012, **24:**2719-2731.

2. Lu ZF, Hofmeister BT, Vollmers C, DuBois RM, Schmitz RJ: **Combining ATAC-seq with nuclei sorting for discovery of cis-regulatory regions in plant genomes.** *Nucleic Acids Research* 2017, **45:**e41.

3. Zhu B, Zhang WL, Zhang T, Liu B, Jiang JM: **Genome-wide prediction and validation of intergenic enhancers in Arabidopsis using open chromatin signatures.** *Plant Cell* 2015, **27:**2415-2426.

4. Wu YF, Zhang WL, Jiang JM: **Genome-wide nucleosome positioning is orchestrated by genomic regions associated with DNase I hypersensitivity in rice.** *PLoS Genet* 2014, **10:**e1004378.

5. Wollmann H, Stroud H, Yelagandula R, Tarutani Y, Jiang D, Jing L, Jamge B, Takeuchi H, Holec S, Nie X, et al: **The histone H3 variant H3.3 regulates gene body DNA methylation in *Arabidopsis thaliana*.** *Genome Biol* 2017, **18:**94.

6. Zhang QZ, Wang D, Lang ZB, He L, Yang L, Zeng L, Li YQ, Zhao C, Huang H, Zhang H, et al: **Methylation interactions in Arabidopsis hybrids require RNA-directed DNA methylation and are influenced by genetic variation.** *Proc Natl Acad Sci USA* 2016, **113:**E4248-E4256.

7. Zemach A, Kim MY, Hsieh PH, Coleman-Derr D, Eshed-Williams L, Thao K, Harmer SL, Zilberman D: **The Arabidopsis nucleosome remodeler DDM1 allows DNA methyltransferases to access H1-containing heterochromatin.** *Cell* 2013, **153:**193-205.

8. Maher KA, Bajic M, Kajala K, Reynoso M, Pauluzzi G, West DA, Zumstein K, Woodhouse M, Bubb K, Dorrity MW, et al: **Profiling of accessible chromatin regions across multiple plant species and cell types reveals common gene regulatory principles and new control modules.** *Plant Cell* 2018, **30:**15-36.

9. Zhang QZ, Li YQ, Xu T, Srivastava AK, Wang D, Zeng L, Yang L, He L, Zhang H, Zheng ZM, et al: **The chromatin remodeler DDM1 promotes hybrid vigor by regulating salicylic acid metabolism.** *Cell Discovery* 2016, **2**.
