# Supplementary material for: Genome-wide MNase hypersensitivity assay unveils distinct classes of open chromatin associated with H3K27me3 and DNA methylation in Arabidopsis thaliana
Source: Genome Biol. 2020 Feb 3;21:24. doi: 10.1186/s13059-020-1927-5 (PMC6996174; doi:10.1186/s13059-020-1927-5)
Supplement: Supplementary file 6 — Review history (DOCX 278 kb) [file 13059_2020_1927_MOESM6_ESM.docx]

Review History

**First round of review**

**Reviewer 1**

**Are you able to assess all statistics in the manuscript, including the appropriateness of statistical tests used?**

Yes, and I have assessed the statistics in my report.

**Comments to author:**

Overall, this is a nice new method that seems to reveal important sequences that have been missed by other assays. This will be of broad interest to those studying chromatin regardless of the species they study. Most importantly, the sMHSs finding is of potential great importance and could offer insight into how polycomb response elements work in plants. However, at this time, there are some critical missing controls that are required prior to publication.

Major comments:

1. Replication of all assays is absolutely required. It doesn't appear that any of the experiments were replicated?

2. The sMHS finding is very interesting and the analyses are fairly convincing. However, a few extra pieces of data should be added to solidify this finding:

a. Figure 5A: Add the ATAC-seq signal to account for the different biases of DNase and Tn5.

b. Add the Tn5 and DNase input signals (activity on naked DNA) to ensure that the lack of ATAC and DNase signal at sMHSs is not an insertion or mapping artifact. The screenshots you've provided throughout the paper indicate that this is not the case, but a non-anecdotal analysis is necessary as well.

c. To figures 5B S3A, add histone H3 ChIP-seq (not targeting specific modification) as an independent signal for nucleosomes which does not rely on nuclease activity. The resolution will be lower than MNase, but the depleted signal in the center of the cMHSs and sMHSs should be visible. The cMHSs are obviously depleted of nucleosomes, and the flanking phased nucleosomes support this. The sMHSs need the independent ChIP-seq support.

d. Add a supplemental figure with at least five screen shots (must be randomly selected) showing sMHSs, ATAC-seq, DNase, and the inputs.

3. Figure 6: add the ATAC and DNase with their inputs (treatment of naked DNA) as two additional columns. This will demonstrate that all of the clusters of sMHSs are valid.

4. There is no explanation in the methods of why 5 groups were selected for k-means. With k-means clustering, it is necessary to add a biological justification and/or an empirical justification, such as an "elbow" plot that demonstrates that k=5 is the best choice.

5. Functional analyses of sMHSs: How did you decide which genes were associated with which MHSs, specifically the intergenic ones? It's not described in the methods.

6. Figure 6: DNA methylation is commonly referred to throughout this study as "repressive", however, ~4,000 genes in Arabidopsis possess DNA methylation in their gene bodies, which are actively transcribed. Therefore, it's not always repressive. CG methylation in isolation is not repressive unless it overlaps the TSS. When it co-occurs with non-CG methylation it is typically repressive. Please split the DNA methylation into CG, CHG and CHH to help clarify this figure and do the same with the values in the main text. Figure S7 shows the DNA methylation are actually mostly expressed, which could indicate many are gene body DNA methylation and not repressed.

7. Figure 7: from your other paper on the enhancer reporter method, are there positive and negative controls that you can add to this figure, to give the reader an idea of what to expect? i.e. what kind of fold-enrichment should we expect for a previously validated enhancer? There are a number of enhancers from Arabidopsis that have already been validated (including the one you show in fig 3c.) These would greatly strengthen the figure. It seems like you should be able to add this information without doing any additional experiments.

8. Figure 8: DNA methylation is not dynamic like H3K27me3, with special exceptions such as in the endosperm or gametes. Therefore the sentences on line 270 should be modified to reflect this point.

9. The text insert in fig. 8d should read "decreased" not increased, correct? Please add a heatmap to support these data. What percent of sMHSs in ddm1 are actually affected. The metaplot shows a bulk change, but it seems like very few are actually affected.

10. Figure 8: could you add a panel to fig 8a which shows the DNase signal from the two tissues? This is required so the signal can be observed instead of just relying on peak-calling.

11. Figure 9b: there is no red square as mentioned in the text. Add a legend for the heatmap colors.

12. We are not convinced that the double-peak pattern around the sMHS centers are biologically relevant. Wouldn't you expect this because the peak at the sMHS won't directly overlap the TF binding site since it's protected from nuclease activity? But since the sMHSs are so small, the double peak pattern is inevitable.

13. It's very possible that the sMHSs which are enriched with H3K27me3 are polycomb response elements. You mention this regarding AZT. This could be done quickly and could add to the paper: take the coordinates for the list of polycomb response elements (from your ref 49, Doris Wagner's paper) and do an overlap analysis. See how the clusters of sMHSs and cMHSs overlap the PREs.

Minor comments:

1. Please add a description of how the data are visualized in Fig. 1b. Are integration sites only shown for ATAC-seq data (1bp of read) or is the entire read shown. If reads are shown for all tracks are the reads the same lengths? If not, this should be amended as it can affect smoothing of peaks in data visualization tools.

2. "These steps were rescued 88,932 reads and these reads were included in further analysis." Could you check that these rescued reads did not disproportionately contribute to the sMHSs? This will ensure that the sMHSs are not mapping artifacts.

3. Please provide more information about the Jazz program. The code for Jazz should be available somewhere so that the results can be replicated by others.

4. The title should be modified to remove "repressive epigenetic marks" as at this time it's not clear if the DNA methylation is associated with gene body DNA methylation or RdDM/silencing methylation.

5. There are numerous references in the supplemental figures. Please ensure they are also listed in the main text references.

**Reviewer 2**

**Are you able to assess all statistics in the manuscript, including the appropriateness of statistical tests used?**

Yes, and I have assessed the statistics in my report.

**Comments to author:**

This is an interesting manuscript. The sequencing of small fragments released by limited MNase digestion provides a complimentary approach to finding regulatory sequences in eukaryotic genomes. Overall, on the whole I found this to be a compelling work that effectively provided evidence for the use of this approach. There are several key concerns that I had as I read the manuscript and authors interpretations. In each case, these could be addressed by further analysis of existing data or clarifying descriptions of results. I will highlight these major significant concerns and provide a list of minor details at the end.

1. A key focus of this work is on the sMHSs sites and the comparison of these sites with the cMHSs sites. In general, the authors seem to rely heavily on peak callers to identify the relevant regions. However, the absence of a peak does not necessarily mean that there isn't some enrichment. Figure 5A was quite useful for showing the lack of DNaseI reads near the sMHS sites. I would appreciate some additional similar plots. I would like to see a plot similar to 5A showing ATAC read depth for cMHSs and sMHSs. In addition, I would like to see a plot similar to this showing read depth for MHS reads. I am curious if the sMHS sites simply tend to have less support than cMHS sites. I do think that the sMHSs do include some unique regions not detected otherwise and are supportable, however, it is not clear to me whether a subset of these may simply represent false positive calls of being MHS sites only. Indeed, when I look at figure 6A it seems the MNase sensitivity is much greater for the cMHSs than for sMHSs.

2. The class 2 sites are quite interesting and I am not sure I agree with the authors assertion that these are accessible regions marked by DNA methylation. Looking at figure 6A it seems that the level of DNA methylation is actually quite low at the actual MHS site (for both cMHS and sMHS) but that there is some methylation nearby. It almost seems that these represent accessible regions near methylated DNA but not necessarily within methylated DNA. I wonder if these might represent potential insulator or boundary element binding sites which would be very interesting. As described below the section on DNA methylation needs additional detail about methods and needs to look at context specific patterns.

3. There are many specific details that are lacking in describing the experiments. I will highlight several of these as they related to the figures:

*	For figure 2 - this could likely be a supplemental figure. What tissue types were used for the ChIPseq?

*	Figure 3B is not a particularly effective presentation of the differences in the length distributions. A density plot with 3 different colors of lines would likely show this data better.

*	In figure 4 - pie charts are not great for showing differences in abundance. I would be better to use stacked bar plots and these could easily be included as a panel in figure 3.

*	In figure 5C I am curious why the trends are so stable for such a large distance, especially for H3K27me3. Is this suggesting that the sMHSs are in the midst of very large H3K27me3 regions?

*	Figure 6 shows a heat map. The units used for each. Modification should be indicated somehow. The DNA methylation analysis in particular is quite unclear. Is this total methylation, CG, CHG, or CHH? I am also confused why there is a directionality for DNA methylation but not other marks. For example, classes 3 and 4 are simply mirror images and likely represent the same biological thing but simply were split based on enrichment on the 5' or 3' sides. I suspect if the authors had used a k-means of 6 they would have found another group similar to class 2 in which there is more methylation on the 5' side of the MHS.

*	Figure 7 seems to suggest only a single replicate was used. Is this true or was this a replicated analysis? Statistical analysis is needed to make the claim that some sMHSs provide functional activity.

*	7B is a bit confusing. Are the two images showing the same leaf or different leaves? If it is the same leaf the middle panel should also be labeled to show which constructs correspond to each peak.

*	In figure 8 (and other analyses) the authors investigate the expression of genes near MHS sites. I could not find any details on how genes were associated with MHS sites. What were the distance and directionality cut-offs?

Specific comments:

Line 82: cue should be cues

Line 109: "The resulted" should probably be "The resulting"

Line 143: Proximately should be approximately

Lines 179-185: This is a really nice example of the power of this technology and I appreciated this highlight

Line 242: Clarify how genes are associated with sMHSs

Line 250; Insert word "the" between showed and same

Line 252-253: I am not fully convinced that this lack of Go terms or expression patterns suggests that class 2 are similar to sMHSs. What is the distance to genes for class 2 sMHSs relative to other groups? I might guess that these would be further away. It is also important to show the profiles of CG, CHG and CHH methylation relative to class 2 sMHSs to show whether all are enriched or not.

Line 263-264: This evidence needs replication and statistical support

Lines 287-293: I am not convinced that this evidence with increased DNaseI sensistivity in ddm1 really shows that these regions are poised for function. If these regions are MHSs because a repressor is bound then why does the same site become more available with loss of methylation?

Line 304: Reversely should probably be Conversely

Line 296-301: I don't understand why the authors are grouping the class 1-5 sMHSs together. Given the differences previously observed it seems that these binding site enrichments should be done for each class separately.

Line 326: Define NDRs (likely nucleosome depleted regions)

**Reviewer 3:**

**Are you able to assess all statistics in the manuscript, including the appropriateness of statistical tests used?**

Yes, and I have assessed the statistics in my report.

**Comments to author:**

The manuscript by Zhao and colleagues describes a new technique, named MH-seq, to identify genomic regions associated with open chromatin in Arabidopsis thaliana. The study is well-designed, the data and analysis are solid. The method will be useful and interesting to the plant chromatin research community. In general, the manuscript is well-written and understandable with the exception of a few issues.

General issues:

Overall, MHSs showed similar overlapping rates with TF-binding sites as peaks derived from DNase-seq and ATAC-seq datasets. Most of peaks identified by DNase-seq or ATAC-seq were covered by MHSs. My question is that if the peak levels of MHSs is also similar with these of DNase-seq and/or ATAC-seq, as peak level is also related to the degree openness, an important parameter of open chromatin that would be good to look at.

What standard was used for judge if a gene was associated with sMHSs and/or cMHSs in Line 198-199?

Line 219-220:"In contrast, sMHSs showed an opposite trend with a high level of H3K27me3 and a low level of H3K27ac (Figure 5C)."As showed in Figure 5C, the H3K27ac level of the flanking regions of sMHSs is indeed lower than that of cMHSs. However, the H3K27ac level of sMHSs is similar as that of cMHSs.

Specific issues:

The legend of Figure 3A is unclear.

Line 175-176:"Furthermore, many MHSs contained multiple peaks with an average length of 47 bp (Figure 3C)." I can not find the information of multiple peaks lengths in Figure 3C.

The category of "transposable element" was included in analysis of genomic features associated with cMHSs and sMHSs (Figure 4) but was excluded in analysis of genomic features associated with MHSs (Figure S2).

Line 189-191: "sMHSs tended to locate at intergenic regions (Figure 4) compared to the MHSs that are covered by DNase-seq or ATAC-seq reads, which are thereafter referred to "common MHSs" (cMHSs). However, the percentage of "intergenic" category is similar for cMHSs and sMHSs as showed in Figure 4.

If "sMHSs with increased CpG methylation in ddm1" in figure 8D should actually be "sMHSs with "decreased" CpG methylation in ddm1"?

**Authors Response**

**Point-by-point responses to the reviewers’ comments:**

Response to comments from Reviewer #1

Overall, this is a nice new method that seems to reveal important sequences that have been missed by other assays. This will be of broad interest to those studying chromatin regardless of the species they study. Most importantly, the sMHSs finding is of potential great importance and could offer insight into how polycomb response elements work in plants. However, at this time, there are some critical missing controls that are required prior to publication.

Major comments:

1. Replication of all assays is absolutely required. It doesn't appear that any of the experiments were replicated?

*Response: Following the reviewer’s comment we have developed a biological replicate of the MH-seq dataset. We call the original MH-seq data as replicate 1 and the new MH-seq data as replicate 2. The two replicates are highly correlated (r=0.82, p-value<2.2×10-16). The replicate 2 data has a higher background noise, which was measured by the fraction of reads in peaks (0.34 for replicate 2 versus 0.61 for replicate 1) and identified less peaks comparing to replicate 1 (44,552 for replicate 2 versus 70,046 for replicate 1). The replicate 2 is developed by a new graduate student with less experience handling chromatin experiments. Nevertheless, 89.7% of the replicate 2 peaks overlapped with replicate 1 data, suggesting that the MH-seq data is reproducible. The different levels of background are common for DNase-seq and MNase-seq techniques as well, caused by tissue quality, different levels of tissue fixation or digestion. We have also conducted replicated experiments of luciferase-based functional assays of selected sMHSs and added stand deviations in Figure 7 (it has been changed as Figure 5 in the revised manuscript).*

2. The sMHS finding is very interesting and the analyses are fairly convincing. However, a few extra pieces of data should be added to solidify this finding:

a. Figure 5A: Add the ATAC-seq signal to account for the different biases of DNase and Tn5.

*Response: ATAC-seq signal has been added in Figure 5A (it has been changed as Figure 3A in the revised manuscript).*

b. Add the Tn5 and DNase input signals (activity on naked DNA) to ensure that the lack of ATAC and DNase signal at sMHSs is not an insertion or mapping artifact. The screenshots you've provided throughout the paper indicate that this is not the case, but a non-anecdotal analysis is necessary as well.

*Response: We have added the Tn5 and DNase input signals over sMHSs and cMHSs in Supplementary Figure S3D. This figure demonstrates that the lack of ATAC or DNase signals at sMHSs is not an insertion or mapping artifact.*

c. To figures 5B S3A, add histone H3 ChIP-seq (not targeting specific modification) as an independent signal for nucleosomes which does not rely on nuclease activity. The resolution will be lower than MNase, but the depleted signal in the center of the cMHSs and sMHSs should be visible. The cMHSs are obviously depleted of nucleosomes, and the flanking phased nucleosomes support this. The sMHSs need the independent ChIP-seq support.

*Response: We thank the Reviewer for this suggestion and have analyzed the ChIP-seq data using an anti-H3 antibody (Abcam, ab1791) (data from Zhang et al. 2018. Nat Commun. 9: 4547). Figure S3E has been added to show the distribution of H3 ChIP-seq reads over cMHSs and sMHSs. The depletion of nucleosomes on sMHSs is now illustrated in this new figure.*

d. Add a supplemental figure with at least five screen shots (must be randomly selected) showing sMHSs, ATAC-seq, DNase, and the inputs.

*Response: Following the Reviewer’s comment we have included five different screenshots in supplementary Figure S4A-E.*

3. Figure 6: add the ATAC and DNase with their inputs (treatment of naked DNA) as two additional columns. This will demonstrate that all of the clusters of sMHSs are valid.

*Response: We have added the ATAC-seq and DNase-seq and their inputs in the clusters of cMHSs and sMHSs. However, the figure looks too bulky with the additional seven columns (4 for ATAC-seq and DNase-seq and three for CG, CHG and CHH methylation). Thus, we instead have developed Supplemental figures S7A and S7B to show the data and have kept Figure 6 unchanged (it has been changed as Figure 4 in the revised manuscript).*

4. There is no explanation in the methods of why 5 groups were selected for k-means. With k-means clustering, it is necessary to add a biological justification and/or an empirical justification, such as an "elbow" plot that demonstrates that k=5 is the best choice.

*Response: An elbow plot showed that the total within-clusters sum of squares (TWSS) changed slower when k=5. This plot has been added as Supplementary Figure S7C. We have modified the relevant text in Methods as “Clustering analysis were conducted using R package “kmeans”. Total within-clusters sum of squares (TWSS) of k from 2 to 16 were calculated. The optimal value of k (k=5) was determined as the smallest value of k that the TWSS changed slower (Figure S7C). The heatmaps of clusters were plotted using R package ‘pheatmap’”.*

5. Functional analyses of sMHSs: How did you decide which genes were associated with which MHSs, specifically the intergenic ones? It's not described in the methods.

*Response: The sMHS-cognate gene is defined as the closest gene located within 1 kb up- or downstream of the sMHS. We have added this information in the Methods as well as in the legend of Figure 8 (now as Figure 6 in the revised manuscript).*

6. Figure 6: DNA methylation is commonly referred to throughout this study as "repressive", however, ~4,000 genes in Arabidopsis possess DNA methylation in their gene bodies, which are actively transcribed. Therefore, it's not always repressive. CG methylation in isolation is not repressive unless it overlaps the TSS. When it co-occurs with non-CG methylation it is typically repressive. Please split the DNA methylation into CG, CHG and CHH to help clarify this figure and do the same with the values in the main text. Figure S7 shows the DNA methylation are actually mostly expressed, which could indicate many are gene body DNA methylation and not repressed.

*Response: We agree with the Reviewer that DNA methylation is not always a “repressive” mark in Arabidopsis depending on whether gene bodies or regulatory regions are methylated. We have modified this description throughout the revised manuscript. We now only point out the association of MHSs with specific marks (DNA methylation and H3K27me3), but not refer these marks as “repressive”.*

*Following the Reviewer’s suggestion we split the DNA methylation into CG, CHG and CHH for analysis. We found CG, CHG and CHH showed similar pattern among different classes. The Class 2 is enriched in DNA methylation in all three (CG, CHG and CHH) context. We have add CHG and CHH in Figure S7A and explained the results in the main text. We have also analyzed the profiles of CG, CHG and CHH methylation on genes in Class 2. Highly expressed genes were associated with CG methylation at gene body and non-expressed genes were associated with CG, CHG and CHH methylation at both gene body and flanking regions, including promoters. A new Figure S9 were provided to show the profiles.*

7. Figure 7: from your other paper on the enhancer reporter method, are there positive and negative controls that you can add to this figure, to give the reader an idea of what to expect? i.e. what kind of fold-enrichment should we expect for a previously validated enhancer? There are a number of enhancers from Arabidopsis that have already been validated (including the one you show in fig 3c.) These would greatly strengthen the figure. It seems like you should be able to add this information without doing any additional experiments.

*Response: We appreciate this suggestion. We have included two previously validated enhancers (L3 and C4) and one negative control (N1) in the modified Figure 7 (it has been changed as Figure 5 in the revised manuscript). L3 and C4 were previously validated as strong enhancers in leave and N1 had no cis-regulatory function.*

8. Figure 8: DNA methylation is not dynamic like H3K27me3, with special exceptions such as in the endosperm or gametes. Therefore the sentences on line 270 should be modified to reflect this point.

*Response: We agree with this comment from the Reviewer. We have modified the sentence and added relevant citations as follow: “DNA methylation is relatively stable epigenetic mark and is less dynamic compared to H3K27me3 during development, with special exceptions such as in the endosperm or gametes (Hsieh et al., 2009; Zemach et al. 2010; Ibarra et al., 2012)”*

9. The text insert in fig. 8d should read "decreased" not increased, correct? Please add a heatmap to support these data. What percent of sMHSs in ddm1 are actually affected. The metaplot shows a bulk change, but it seems like very few are actually affected.

*Response: Yes, the text insert in fig. 8d should read “decreased”. We analyzed the effects of ddm1 on chromatin openness and gene expression in class 2 based on this comment as well as a similar comment from Reviewer 2. We found that very few genes (3.2%) in class 2 showed changes of expression levels between wild type and ddm1 mutant. Furthermore, chromatin openness is also largely unchanged in class 2 between wild type and ddm1 mutant. Therefore, we have decided to remove figure 8d and added the updated analysis in the revised manuscript.*

10. Figure 8: could you add a panel to fig 8a which shows the DNase signal from the two tissues? This is required so the signal can be observed instead of just relying on peak-calling.

*Response: We have added the DNase signals of the two tissues as suggested, which confirmed the peak-calling results that the sMHSs show a higher level of DNase I sensitivity in flower buds. The original Figure 8 is changed to Figure 6 in the revised manuscript.*

11. Figure 9b: there is no red square as mentioned in the text. Add a legend for the heatmap colors.

*Response: We are sorry for the error. A color key has been added in the figure. The red square is no longer needed since the description of “double-peak distribution” has been removed (please see our response to comment #12).*

12. We are not convinced that the double-peak pattern around the sMHS centers are biologically relevant. Wouldn't you expect this because the peak at the sMHS won't directly overlap the TF binding site since it's protected from nuclease activity? But since the sMHSs are so small, the double peak pattern is inevitable.

*Response: We respect the Reviewer’s concern on our explanation. We have decided to delete the context of the double-peak pattern.*

13. It's very possible that the sMHSs which are enriched with H3K27me3 are polycomb response elements. You mention this regarding AZT. This could be done quickly and could add to the paper: take the coordinates for the list of polycomb response elements (from your ref 49, Doris Wagner's paper) and do an overlap analysis. See how the clusters of sMHSs and cMHSs overlap the PREs.

*Response: We appreciate this comment from the Reviewer. We have analyzed the overlap between the 170 PREs in ref 49 and the sMHSs that are enriched with H3K27me3. We found that 11 PREs overlap with sMHSs. We then analyzed the distribution of MH-seq reads, DNase-seq reads, MNase-seq reads and H3K27me3 on the 170 PREs. The centers of these PREs showed background levels of MH-seq and DNase-seq signals but higher levels of nucleosomes and H3K27me3 marks (the figure below. Please find the figure in the uploaded supplemental material.). Thus, it is likely that these PREs in the tissue we studied are well occupied by nucleosomes and are not accessible to MNase.*

Minor comments:

1. Please add a description of how the data are visualized in Fig. 1b. Are integration sites only shown for ATAC-seq data (1bp of read) or is the entire read shown. If reads are shown for all tracks are the reads the same lengths? If not, this should be amended as it can affect smoothing of peaks in data visualization tools.

*Response: We have re-draw the Fig. 1b. For ATAC-seq and all other data, we generated the read coverage track using the center of reads at 1-bp resolution so that all the data now have the same smoothing level.*

2. "These steps were rescued 88,932 reads and these reads were included in further analysis." Could you check that these rescued reads did not disproportionately contribute to the sMHSs? This will ensure that the sMHSs are not mapping artifacts.

*Response: We have re-analyzed the data and found that these reads don’t contribute to the peak calling as the peaks are unchanged by removing these reads. Indeed, the 88,932 reads only account for a very low percentage of the total reads (0.08%). We have removed these reads in the revision to avoid confusing readers.*

3. Please provide more information about the Jazz program. The code for Jazz should be available somewhere so that the results can be replicated by others.

*Response: We have added a Supplemental Method to provide detailed description of the Jazz algorithm. The code has also been deposited in github (https://github.com/zhangtaolab/Jazz/).*

4. The title should be modified to remove "repressive epigenetic marks" as at this time it's not clear if the DNA methylation is associated with gene body DNA methylation or RdDM/silencing methylation.

*Response: Following the Reviewer’s suggestion we have changed the title as “Genome-wide MNase hypersensitivity assay unveils distinct classes of open chromatin associated with H3K27me3 and DNA methylation in Arabidopsis thaliana”.*

5. There are numerous references in the supplemental figures. Please ensure they are also listed in the main text references.

*Response: We have made the references in the supplemental figures and tables listed in the main references.*

Response to comments from Reviewer #2

Reviewer #2: This is an interesting manuscript. The sequencing of small fragments released by limited MNase digestion provides a complimentary approach to finding regulatory sequences in eukaryotic genomes. Overall, on the whole I found this to be a compelling work that effectively provided evidence for the use of this approach. There are several key concerns that I had as I read the manuscript and authors interpretations. In each case, these could be addressed by further analysis of existing data or clarifying descriptions of results. I will highlight these major significant concerns and provide a list of minor details at the end.

1. A key focus of this work is on the sMHSs sites and the comparison of these sites with the cMHSs sites. In general, the authors seem to rely heavily on peak callers to identify the relevant regions. However, the absence of a peak does not necessarily mean that there isn't some enrichment. Figure 5A was quite useful for showing the lack of DNaseI reads near the sMHS sites. I would appreciate some additional similar plots. I would like to see a plot similar to 5A showing ATAC read depth for cMHSs and sMHSs. In addition, I would like to see a plot similar to this showing read depth for MHS reads. I am curious if the sMHS sites simply tend to have less support than cMHS sites. I do think that the sMHSs do include some unique regions not detected otherwise and are supportable, however, it is not clear to me whether a subset of these may simply represent false positive calls of being MHS sites only. Indeed, when I look at figure 6A it seems the MNase sensitivity is much greater for the cMHSs than for sMHSs.

*Response: We thank the Reviewer for the great comment. We have added the ATAC-seq read distribution over cMHSs and sMHSs in Figure 5A (it has been changed as Figure 3A in the revised manuscript). The ATAC-seq shows a similar pattern as DNase-seq. A supplementary figure S3D is also added to show the MH-seq reads over cMHSs and sMHSs. The cMHSs and sMHSs have similar depth of MH-seq, except that sMHSs has a narrower read distribution.*

*The MNase sensitivity of sMHSs is only slightly lower than cMHSs (see the Figure below.Please find the figure in the uploaded supplemental material.). The difference of MNase sensitivity for the cMHSs and sMHSs in Figure 6 is not well displayed due to the binning process at the two different types of MHSs. The heatmap shows the average read depth in each bin, which is calculated as the number of reads divided by the size of the bin. As sMHSs is short, the bin spanning the sMHSs often includes the flanking regions, which lead to a lower read depth. The figure bellow shows the average read depth of different sizes of bins. As bin size increases, the average read depth reduces for sMHSs, but it has little impact on cMHSs. We have added the following sentence in the legend of Figure 6A (changed to Figure 4 in the revised manuscript): “genomic regions up- and downstream 1 kb of centers of MHSs were divided into 40 bins. The average read depth in each bin was calculated. The average read depth from each data set was then scaled from 0 to 1 and plotted as a heatmap.”*

2. The class 2 sites are quite interesting and I am not sure I agree with the authors assertion that these are accessible regions marked by DNA methylation. Looking at figure 6A it seems that the level of DNA methylation is actually quite low at the actual MHS site (for both cMHS and sMHS) but that there is some methylation nearby. It almost seems that these represent accessible regions near methylated DNA but not necessarily within methylated DNA. I wonder if these might represent potential insulator or boundary element binding sites which would be very interesting. As described below the section on DNA methylation needs additional detail about methods and needs to look at context specific patterns.

*Response: We are thankful to these comments. The Reviewer is correct that the body of MHSs are not methylated. The immediate flanking region of the class 2 MHSs are methylated. We have modified the relevant text in the revised manuscript.*

3. There are many specific details that are lacking in describing the experiments. I will highlight several of these as they related to the figures:

* For figure 2 - this could likely be a supplemental figure. What tissue types were used for the ChIPseq?

*Response: Following the Reviewer’s suggestion we have moved figure 2 as a supplemental figure (Figure S2B) and added a column of tissue type in Table S1.*

* Figure 3B is not a particularly effective presentation of the differences in the length distributions. A density plot with 3 different colors of lines would likely show this data better.

*Response: We have changed the plot (it has been changed as Figure 2B) in the revised manuscript) to be a density plot as the Reviewer suggested.*

* In figure 4 - pie charts are not great for showing differences in abundance. I would be better to use stacked bar plots and these could easily be included as a panel in figure 3.

*Response: Figure 4 has been changed to bar plots and included as part of Figure 3 (it has been changed as Figure 2 in the revised manuscript).*

* In figure 5C I am curious why the trends are so stable for such a large distance, especially for H3K27me3. Is this suggesting that the sMHSs are in the midst of very large H3K27me3 regions?

*Response: The H3K27me3 mark tends to form broad peaks in the Arabidopsis genome. The average length of the H3K27me3 peaks is 1.6 kb. We found that the H3K27me3 peaks overlapped with sMHSs is 3.3 kb, which is larger than the genome average.*

* Figure 6 shows a heat map. The units used for each. Modification should be indicated somehow. The DNA methylation analysis in particular is quite unclear. Is this total methylation, CG, CHG, or CHH? I am also confused why there is a directionality for DNA methylation but not other marks. For example, classes 3 and 4 are simply mirror images and likely represent the same biological thing but simply were split based on enrichment on the 5' or 3' sides. I suspect if the authors had used a k-means of 6 they would have found another group similar to class 2 in which there is more methylation on the 5' side of the MHS.

*Response: We have added the color key for the Figure 6 (changed to Figure 4 in the revised manuscript). The following sentence was added in the figure legend: “Genomic regions up- and downstream 1kb of MHSs were divided into 40 bins. The average read depth and DNA methylation level in each bin were calculated. The average read depth from each dataset was scaled from 0 to 1 and plotted as a heatmap.”*

*We found that class 2 is flanked by sequences associated with all three types of DNA methylation (CG, CHG and CHH). Furthermore, the patterns of CG, CHG and CHH methylation are similar among different classes. Therefore, we decided to only show CG methylation in Figure 4. A supplementary figure S7A was provided to show the DNA methylation in all three context.*

*The cluster number was determined using elbow method. We tested k from 2 to 16 and found that k=5 is the optimal number of clusters and is the best to classify the data. A supplementary figure S7C is provided.*

* Figure 7 seems to suggest only a single replicate was used. Is this true or was this a replicated analysis? Statistical analysis is needed to make the claim that some sMHSs provide functional activity.

*Response: Following the Reviewer’s comments we have conducted replicated experiments of luciferase-based functional assays of selected sMHSs. We have performed the t-test of the data and the results have been added in the figure. The original Figure 7 has been changed to Figure 5 in the revised manuscript.*

* 7B is a bit confusing. Are the two images showing the same leaf or different leaves? If it is the same leaf the middle panel should also be labeled to show which constructs correspond to each peak.

*Response: We are sorry for overlooking the labeling of the Figure. The two images show the result from the same leaf. We have added labels in the middle panel to show the construct of each peak.*

* In figure 8 (and other analyses) the authors investigate the expression of genes near MHS sites. I could not find any details on how genes were associated with MHS sites. What were the distance and directionality cut-offs?

*Response: The MHS-cognate gene is defined as the closest gene located within 1 kb up- or downstream of the MHS. We have added this information in the legend of Figure 8 (changed to Figure 6 in the revised manuscript) as well as in Methods.*

Specific comments:

Line 82: cue should be cues

Line 109: "The resulted" should probably be "The resulting"

Line 143: Proximately should be approximately

*Response: We have changed the words as suggested.*

Lines 179-185: This is a really nice example of the power of this technology and I appreciated this highlight.

*Response: thank you.*

Line 242: Clarify how genes are associated with sMHSs

*Response: Please see our response to Reviewer’s comments “In figure 8…”*

Line 250; Insert word "the" between showed and same

*Response: We have inserted “the” in the sentence.*

Line 252-253: I am not fully convinced that this lack of Go terms or expression patterns suggests that class 2 are similar to sMHSs. What is the distance to genes for class 2 sMHSs relative to other groups? I might guess that these would be further away. It is also important to show the profiles of CG, CHG and CHH methylation relative to class 2 sMHSs to show whether all are enriched or not.

*Response: We are grateful to the Reviewer for this comment and have deleted the sentence in Line 252-253. The distances to genes for MHSs in each classes have been added in Figure S7A. Class 2 sMHSs are indeed further away from genes than other classes. The profiles of CG, CHG and CHH methylation have been added in the supplementary Figure S7 (also see our response to similar comments from Reviewer 1).*

Line 263-264: This evidence needs replication and statistical support

*Response: Replications and statistical support have been added in the revised manuscript and in Figure 7 (it has been changed to Figure 5 in the revised manuscript).*

Lines 287-293: I am not convinced that this evidence with increased DNaseI sensistivity in ddm1 really shows that these regions are poised for function. If these regions are MHSs because a repressor is bound then why does the same site become more available with loss of methylation?

*Response: We have re-analyzed DNA methylation data as Reviewer 1 also suggested. We found that although a bulk change was observed in the aggregated plot, very few MHSs were affected in ddm1. Therefore, we have decided to remove the analysis.*

Line 304: Reversely should probably be Conversely

*Response: Changed as suggested.*

Line 296-301: I don't understand why the authors are grouping the class 1-5 sMHSs together. Given the differences previously observed it seems that these binding site enrichments should be done for each class separately.

*Response: We agree that each class probably show different binding site enrichments. However, separating the classes reduce the number of sMHSs significantly, which then limits the power of motif discovery and produce more false positive results. This is why we chose to bulk sMHSs all together and focus on comparing different binding sites between sMHSs and cMHSs.*

Line 326: Define NDRs (likely nucleosome depleted regions)

*Response: The definition of NDRs (nucleosome depleted regions) has been added.*

Response to comments from Reviewer #3

The manuscript by Zhao and colleagues describes a new technique, named MH-seq, to identify genomic regions associated with open chromatin in Arabidopsis thaliana. The study is well-designed, the data and analysis are solid. The method will be useful and interesting to the plant chromatin research community. In general, the manuscript is well-written and understandable with the exception of a few issues.

General issues:

Overall, MHSs showed similar overlapping rates with TF-binding sites as peaks derived from DNase-seq and ATAC-seq datasets. Most of peaks identified by DNase-seq or ATAC-seq were covered by MHSs. My question is that if the peak levels of MHSs is also similar with these of DNase-seq and/or ATAC-seq, as peak level is also related to the degree openness, an important parameter of open chromatin that would be good to look at.

*Response: Following the Reviewer’s comments we analyzed the peak levels of cMHSs, which can be identified by both DNase-seq and ATAC-seq. The peak levels were measured as the reads per million, which is commonly used to measure gene expression levels (Wagner et al., 2012). We found that the peak levels of MH-seq, DNase-seq and ATAC-seq were well correlated. The correlation of peak levels between MH-seq and DNase-seq is 0.38 (p-value <1×10-16, Spearman's rank correlation coefficient). The correlation of peak levels between MH-seq and ATAC-seq is 0.25 (p-value <1×10-16, Spearman's rank correlation coefficient). The correlation of peak levels between DNase-seq and ATAC-seq is 0.42 (p-value <1×10-16, Spearman's rank correlation coefficient).*

What standard was used for judge if a gene was associated with sMHSs and/or cMHSs in Line 198-199?

*Response: The sMHS-cognate genes are defined as genes located within 1 kb up- and downstream of the sMHSs. We have added this information in the Methods (also see our response to comment #5 from Reviewer 1).*

Line 219-220:"In contrast, sMHSs showed an opposite trend with a high level of H3K27me3 and a low level of H3K27ac (Figure 5C)."As showed in Figure 5C, the H3K27ac level of the flanking regions of sMHSs is indeed lower than that of cMHSs. However, the H3K27ac level of sMHSs is similar as that of cMHSs.

*Response: The H3K27ac level of sMHSs is similar as that of cMHSs, which is due to the lack of nucleosomes at the centers of both sMHSs and cMHSs. The H3K27ac levels at the center of cMHSs and sMHSs represent the signal background. Previous studies also showed that H3K27ac is enriched at the flanking regions of open chromatin region and is nearly depleted at the center of open chromatin regions in plants (Zhang et al. 2012, Genome Research).*

Specific issues:

The legend of Figure 3A is unclear.

*Response: We are sorry for the confusing legend. We have added additional explanation in this legend: “The rows of the matrix represent the source features, while the columns represent the linked features. The percentage in each cell at the intersection of a row and column represents the overlapping rate between the source feature and the linked feature. For example, the cell at the intersection of MHS row and the DHS (single cut) column indicates that 61% of MHSs overlapped with DHSs”.*

Line 175-176:"Furthermore, many MHSs contained multiple peaks with an average length of 47 bp (Figure 3C)." I can not find the information of multiple peaks lengths in Figure 3C.

*Response: We have added a Figure S3C to show the distribution of the length of peaks within MHSs.*

The category of "transposable element" was included in analysis of genomic features associated with cMHSs and sMHSs (Figure 4) but was excluded in analysis of genomic features associated with MHSs (Figure S2).

*Response: We have added the category of “transposable elements” in Figure S2. The definition of “transposable elements” and “intergenic” has been added in the legend. In addition, the original Figure 4 was changed to stacked bar plot and is now included in the new Figure 2.*

Line 189-191: "sMHSs tended to locate at intergenic regions (Figure 4) compared to the MHSs that are covered by DNase-seq or ATAC-seq reads, which are thereafter referred to "common MHSs" (cMHSs). However, the percentage of "intergenic" category is similar for cMHSs and sMHSs as showed in Figure 4.

*Response: The intergenic regions here are referred as genomic regions that are at least 3 kb away from genes. In addition, the transposable elements were also considered as intergenic regions. Thus, the percentage of "intergenic" category of sMHSs is higher than cMHSs. We have modified the sentence Line 189-192 as follow for clarity: “sMHSs tended to locate at intergenic regions (at least 3 kb away from any annotated genes, as well as those associated with TEs) (Figure 2C) compared to the MHSs that are covered by DNase-seq or ATAC-seq reads, which are thereafter referred to “common MHSs” (cMHSs). Interestingly, inspecting the MHSs located in the intergenic regions revealed that 18% of the sMHSs were associated with transposable elements (TEs), comparing 4% of cMHSs associated with TEs”.*

If "sMHSs with increased CpG methylation in ddm1" in figure 8D should actually be "sMHSs with "decreased" CpG methylation in ddm1"?

*Response: Yes, the text in figure 8D should read “decreased”, we are sorry for the typo (please also see our response to comment # 9 from Reviewer 1)*

**Second round of review**

**Reviewer 1**

I appreciate the authors taking the time to address the original reviews. The revised manuscript is excellent. It’s a minor point, but the failure to highly reproduce the MH-seq data in the second replicate is a little worrisome. If the lab that developed the technique is unable to easily reproduce the method with high confidence, then how challenging will it be for other labs to adopt this novel methodology. Maybe a comment or two about the difficulty of this assay could be included somewhere in the manuscript.

**Reviewer 2**

The authors have done a nice job of addressing my comments. I find this to be an interesting study. I had a couple of minor issues I noted in the revised version with a couple suggestions for analyses that should not be too difficult.

Page 4 Line 32-33 should probably refer to replicate 2 (currently reads “although replicate has a higher percentage…”)

The analyses in Figure S2B are worthwhile and helpful. These should be repeated for cMHS and sMHS sites to look at both of these sets separately. Later in the manuscript TF binding sites in cMHS and sMHS are investigated and they should look at the ChIP-seq data for these, not just predicted binding sites.

Page 5 Line 40; In this section you note that often there are a cluster of multiple MHS peaks within a singe DHS or ATAC-Seq peak. When you look at the sMHS peaks are these more often single peaks rather than clusters? In other words, are these often just one region between nucleosomes or a collection of multiple regions compared to the cMHSs?

Page 6 Line 30-32: The authors state that “cMHSs were associated with a low level of H3K27me3 and high level of H3K27ac” I would suggest altering the phrasing a bit. The cMHSs actually do not have any enrichment/depletion. Instead, the regions flanking cMHSs are enriched/depleted for these marks. This should be clarified.

Page 7 line 11-12; Is there an issue with focusing on sMHSs within 1kb of genes? In the previous paragraph is was highlighted that sMHSs are often in intergenic regions further from genes. By focusing only on those close to genes are you biasing the results?

Page 8 Lines 15-30. This section is poorly written and needs editing. For example, “Genes with body methylation only was dominated by…” The word context should often be plural in this section (contexts)

Overall, I am still not all that convinced by the interpretation of the class 2 sMHSs. My impression is that these are open regions right at the edge of methylated regions. I would encourage the authors to look at some of the ChIPseq data for PolIV components. My prediction is that these may actually reflect open regions right at the edge of heterochromatin being used for PolIV access.

**Authors Response**

**Point-by-point responses to the reviewers’ comments:**

Response to comments from Reviewer #1

I appreciate the authors taking the time to address the original reviews. The revised manuscript is excellent. It’s a minor point, but the failure to highly reproduce the MH-seq data in the second replicate is a little worrisome. If the lab that developed the technique is unable to easily reproduce the method with high confidence, then how challenging will it be for other labs to adopt this novel methodology. Maybe a comment or two about the difficulty of this assay could be included somewhere in the manuscript.

*Response: We are grateful to the Reviewer for his/her understanding about the reproducibility of the MH-seq data. Our second MH-seq data showed a higher background than the first data, which is often caused by the tricky chromatin-based techniques. Following the Reviewer suggestion we have added several sentences in the Methods section to remark the background and quality issue of different MH-seq datasets, which will be useful for the readers.*

*We have recently developed MH-seq datasets in soybean and have conducted a comparative analysis with a recently published ATAC-seq dataset of soybean (Lu et al. 2019 Nat. Plants 5: 1250-59). We identified 36,882 ATAC-seq peaks and 134,780 MHSs, respectively (both datasets were developed using leaf tissue of soybean cultivar Williams 82). Approximately 60% of ATAC-seq peaks overlap with MHSs. Strikingly, nearly 79% of the MHSs are not covered by ATAC-seq peaks. Thus, ATAC-seq misses even more open chromatin regions in soybean compared to Arabidopsis. A few screen shots of the ATAC-seq/MH-seq comparison in soybean are provided in the uploaded supplemental file “Response to 2nd Review”.*

Response to comments from Reviewer #2

The authors have done a nice job of addressing my comments. I find this to be an interesting study. I had a couple of minor issues I noted in the revised version with a couple suggestions for analyses that should not be too difficult.

Page 4 Line 32-33 should probably refer to replicate 2 (currently reads “although replicate has a higher percentage…”)

*Response: We have modified the sentence as “although the second dataset has a higher percentage…” to clarify the description of the replicated data.*

The analyses in Figure S2B are worthwhile and helpful. These should be repeated for cMHS and sMHS sites to look at both of these sets separately. Later in the manuscript TF binding sites in cMHS and sMHS are investigated and they should look at the ChIP-seq data for these, not just predicted binding sites.

*Response: We have added a stacked bar plot (Figure S2C) to show the overlap rate between the TF ChIP-seq peaks and cMHSs/sMHSs, respectively. We found that the majority of ChIP-seq peaks overlap with cMHSs, which is likely due to the differential chromatin status and different sizes of cMHSs/sMHSs.*

*We prefer to use predicted binding sites, instead of using ChIP-seq data, for two reasons: First, sMHSs are generally not well captured by ChIP-seq experements (Figure S2C). Second, the predicted binding sites represent the full binding potential of transcription factors as they are not restricted to a specific tissue and/or development stage.*

Page 5 Line 40; In this section you note that often there are a cluster of multiple MHS peaks within a singe DHS or ATAC-Seq peak. When you look at the sMHS peaks are these more often single peaks rather than clusters? In other words, are these often just one region between nucleosomes or a collection of multiple regions compared to the cMHSs?

*Response: This is a great question. We observed both single sMHS and multiple sMHSs that are located close to each other. However, the distance between neighboring sMHSs is greater than those of neighboring cMHSs/MHSs (a figure is provided in the uploaded supplemental file “Response to 2nd Review”). Therefore, sMHSs tend to be single peaks more frequently than cMHSs/MHSs.*

Page 6 Line 30-32: The authors state that “cMHSs were associated with a low level of H3K27me3 and high level of H3K27ac” I would suggest altering the phrasing a bit. The cMHSs actually do not have any enrichment/depletion. Instead, the regions flanking cMHSs are enriched/depleted for these marks. This should be clarified.

*Response: We thank the reviewer to catch this, and have changed the sentence accordingly.*

Page 7 line 11-12; Is there an issue with focusing on sMHSs within 1kb of genes? In the previous paragraph is was highlighted that sMHSs are often in intergenic regions further from genes. By focusing only on those close to genes are you biasing the results?

*Response: Since enhancers can regulate genes in long distance, it would be more error-prone to assign a distal sMHS to a putative cognate gene. In addition, genes related (putatively) to a distal sMHS are often associated with cMHSs, which complicates the analysis. Thus, we decided to focus on the sMHSs within 1kb of genes.*

Page 8 Lines 15-30. This section is poorly written and needs editing. For example, “Genes with body methylation only was dominated by…” The word context should often be plural in this section (contexts)

*Response: We appreciate the comments and have edited text in this the section accordingly.*

Overall, I am still not all that convinced by the interpretation of the class 2 sMHSs. My impression is that these are open regions right at the edge of methylated regions. I would encourage the authors to look at some of the ChIPseq data for PolIV components. My prediction is that these may actually reflect open regions right at the edge of heterochromatin being used for PolIV access.

*Response: We analyzed the position of class 2 MHSs relative to methylated regions. Both sMHSs and cMHSs in class 2 were enriched at the edges of methylated regions, although sMHSs also existed within methylated regions (please see Figure A in the uploaded supplemental file “Response to 2nd Review”). Analysis of the distribution of polIV around MHSs showed that the flanking region of class 2 cMHSs were enriched for polIV, while class 2 sMHSs showed similar level of polIV enrichment to the background. (please see Figure B in the uploaded supplemental file "reponse to 2nd Review").*
